# Integrating multivariate selection indices with weighted rank aggregation to identify drought-tolerant *Populus simonii* × *P. nigra* F_1_ progenies

**DOI:** 10.1186/s12870-025-08054-9

**Published:** 2026-01-19

**Authors:** Zhengyuan Zhou, Mingrong Cao, Dongxu Jia, Chenggong Liu, Qinjun Huang, Jinhua Li

**Affiliations:** 1 State Key Laboratory of Tree Genetics and Breeding, Research Institute of Forestry, Chinese Academy of Forestry, Beijing, 100091 China; 2 Key Laboratory of Tree Breeding and Cultivation, State Forestry and Grassland Administration, Beijing, 100091 China

**Keywords:** *P*. *simonii* × *P*. *nigra*, Drought tolerance, Multivariate selection, Smith-Hazel index, MGIDI, Weighted Rank Aggregation (WRA)

## Abstract

**Background:**

Breeding drought-tolerant poplar cultivars necessitates efficient selection strategies that can simultaneously improve multiple traits. This study evaluated the integration of multivariate selection indices with Weighted Rank Aggregation (WRA) to identify superior genotypes in hybrid poplar progenies.

**Methods:**

We assessed 372 F_1_ progenies from three families of *Populus simonii* × *P. nigra* under controlled drought stress and well-watered conditions. Data on 16 growth, leaf, and photosynthetic traits were analyzed using four multivariate indices: the Smith-Hazel Index (SHI), FAI-BLUP, and two Multi-Trait Genotype-Ideal Genotype Distance Index (MGIDI) variants. The rankings were integrated using WRA.

**Results:**

Genetic parameters revealed high heritability for key growth traits. The selection indices exhibited divergent focus, with SHI showing strong directional selection for growth but sensitivity to multicollinearity, while FAI-BLUP and MGIDI enabled more balanced multi-trait improvements. Most indices were weakly correlated (Spearman’s |r| < 0.2), indicating complementary information. Venn analysis identified genotypes (e.g., C4‑246, E4‑70) performing consistently across multiple indices. The final WRA integration selected robust genotypes, including C2‑65, C4‑210, E4‑115, and E4‑70, which combine drought tolerance with desirable growth and physiological characteristics.

**Conclusions:**

Integrating multiple selection indices with WRA provides a powerful and reliable strategy for selecting drought-tolerant poplar genotypes at the seedling stage. This approach effectively balances genetic gains across traits, enhancing the efficiency of breeding programs for stress resilience.

**Supplementary Information:**

The online version contains supplementary material available at 10.1186/s12870-025-08054-9.

## Introduction

Poplars (*Populus* spp.) are among the world’s most critical fast-growing timber trees, valued for their rapid growth and wide adaptability [1, 2]. However, their productivity is severely threatened by increasing drought and water scarcity driven by climate change, particularly in arid and semi-arid regions [3–6]. Breeding drought-tolerant cultivars is therefore essential to ensure the sustainability of poplar plantations [7–9]. A major challenge in this endeavor is that drought tolerance is a complex trait governed by multiple physiological mechanisms, making selection based on a single or a few traits ineffective [10–13]. Consequently, numerous growth, leaf, and physiological traits have been implicated in drought response [14, 15]. However, a consensus on which traits are definitive indicators is lacking, as studies often employ disparate subsets of traits [16]. Therefore, employing reliable methods that can integrate multiple traits into a comprehensive selection index is crucial for advancing poplar breeding.

The pursuit of multi-trait selection has led to the development of several statistical indices. The Smith-Hazel Index (SHI) is a classical method that enables directional selection but is often compromised by multicollinearity among traits and the subjective assignment of economic weights [17–19]. To address these limitations, more robust indices have been introduced. The Factor Analytic Index combined with Best Linear Unbiased Prediction (FAI-BLUP) effectively disentangles genotypic values from environmental noise, facilitating balanced genetic gains [20–22]. Similarly, the Multi-Trait Genotype-Ideal Genotype Distance Index (MGIDI) circumvents the need for economic weights and employs factor analysis to provide a holistic assessment of genotype performance [23, 24]. While these indices have been successfully applied in crops like soybean and barley [21, 24–26], and their combined use has been explored in black bean and barley breeding [21, 27], a critical gap remains. The integration of their divergent rankings into a unified decision-making framework, such as via Weighted Rank Aggregation (WRA), has rarely been reported in plant breeding and is entirely unexplored for drought-tolerant poplar.

The hybrid offspring of *P. simonii* and *P. nigra* represent ideal material to address this challenge. *P. simonii* exhibits remarkable tolerance to arid and infertile soils, while *P. nigra* contributes rapid growth and high yield [28, 29]. Their hybrids combine these advantageous traits, demonstrating superior growth and stress resilience, making them prime candidates for afforestation in Northern China’s demanding environments [30–32]. Based on this rationale, the present study was designed with the following objectives: (1) To evaluate and compare the reliability of four multivariate selection indices (SHI, FAI-BLUP, MGIDI_BLUP, MGIDI_LWindex) for identifying superior genotypes. (2) To analyze the correlations and consensus among these indices. (3) To integrate the indices using Weighted Rank Aggregation (WRA) to select robust drought-tolerant poplar progenies with balanced performance across growth and physiological traits.

## Materials and methods

### Plant materials

We used a total of 372 F_1_ progenies from three hybrid families of *Populus simonii* × *P. nigra*. The crosses were made between two female clones (*P. simonii* cl. ‘1-XY’ and *P. pseudo-simonigra* cl. ‘ZL-3’) from the Tongliao Gene Bank (Inner Mongolia, China), which are tolerant to poor soils [33], and two male clones (*P. nigra* cl. ‘N188’ and ‘N139’) from the Italian Gene Bank of *P. nigra*, known for fast growth and superior wood quality [34]. The three families of interspecific crosses were identified as follows: *P. simonii* cl. ‘1-XY’ × *P. nigra* cl. ‘N139’ (‘1-XY’×‘N139’), *P. simonii* cl. ‘1-XY’ × *P. nigra* cl. ‘N188’ (‘1-XY’×‘N188’), and *P. pseudo-simonigra* cl. ‘ZL-3’ × *P. nigra* cl. ‘N188’ (‘ZL-3’×‘N188’).

### Potting experiment in greenhouse

The experiment was conducted in a greenhouse at the Chinese Academy of Forestry, Beijing, China. Stem cuttings were collected in February 2024 and potted in early March in plastic pots (9 cm in diameter, 18 cm in height) containing a 2:1 (*v/v*) mixture of peat and perlite (Seedling substrate, Shanghai Branch of Pindstrup Horticulture Co., Ltd., Ryomgaard, Denmark). The substrate had a pH of 5.5 and minimal initial fertilizer content. Plants were grown under natural sunlight without shading, and the greenhouse temperature was maintained at 25 ± 3 °C throughout the experiment.

After approximately 40 days of establishment, seedlings were subjected to two water treatments for a duration of 30 days. The soil moisture thresholds were defined with reference to established poplar physiology research, wherein approximately 75% of field water capacity represents well-watered conditions, and approximately 40% induces severe physiological drought stress [35]. Accordingly, the treatments were implemented as follows:

1. Normal-watered (NW): Soil relative water content was maintained at 75–80%.
2. Low-level watered (LW): Soil relative water content was maintained at 35–40%.

The soil relative water content was calculated as [36]:

$$\begin{aligned} &\mathrm{Soil}\;\mathrm{relative}\;\mathrm{water}\;\mathrm{content}\;\\&=\;\left(\mathrm{Soil}\;\mathrm{gravimetric}\;\mathrm{water}\;\mathrm{content}\; \right. \\& \left. \quad /\;\mathrm{Field}\;\mathrm{water}\;\mathrm{capacity}\right)\;\times\;100\% \end{aligned}$$

The experiment followed a completely randomized block design with three replications, each consisting of a single plant per experimental unit. Each treatment group comprised 186 plants, resulting in a total of 372 plants. Field water capacity was determined according to the method described by Wilcox [37]. Thereafter, soil relative water content was maintained within the predetermined ranges by measuring pot weights every other day and adding water accordingly.

### Measurements of traits

Plant morphological and physiological traits were measured throughout the experiment. Initial plant height (H1, cm) and base diameter (D1, mm) were recorded in mid-April, prior to the imposition of drought stress, using a graduated ruler and a digital caliper, respectively.

During the peak growing season (July 10–20), traits were measured on one fully expanded, mature leaf collected from the 5th to 7th node from the apex of each plant. Leaf area (LA, cm^2^) was determined using the LeafByte smartphone application [38]. We then measured leaf fresh weight (FW, g), leaf turgid weight (TW, g), and leaf dry weight (LDW, g) following the standard protocol for these specific traits [39]. FW was recorded immediately after excision. Leaves were then immersed in deionized water for 24 h in darkness until fully saturated; after blotting surface moisture, TW was measured. Samples were then oven-dried at 105 °C for 20 min followed by drying at 80 °C to constant weight to obtain LDW. Leaf mass per area (LMA, g∙cm^-2^) was calculated as $\mathrm{LA}/\mathrm{LDW} (\mathrm{FW-LDW})/(\mathrm{TW} - \mathrm{LDW}) \times 100$. All weights were measured using an electronic balance.

Photosynthetic parameters were measured on clear mornings between 9:00 and 12:00 using a Li-6400XT portable photosynthesis system (Li-COR Inc., USA). The measurements were conducted under controlled conditions: chamber temperature set at 25 °C, photosynthetic photon flux density at 1200 µmol∙m^-2^s^-1^, and CO_2_ concentration at 400 ppm [35]. The directly recorded parameters included transpiration rate (TR, mmol∙m^-2^s^-1^), stomatal conductance (GA, mmol∙m^-2^s^-1^), net photosynthetic rate (PN, µmol∙m^-2^s^-1^), and intercellular CO₂ concentration (CI, µmol∙mol^-1^). Derived parameters were calculated as follows: stomatal limitation value (LS, %) $\:\mathrm{=\:1}\mathrm{-}\mathrm{CI/G}\mathrm{S}\times\:100$, and instantaneous water use efficiency (WUE, mmol∙mol^-1^) $\:=\:\mathrm{P}\mathrm{N}/\mathrm{T}\mathrm{R}$.

The final plant height (H2, cm) and base diameter (D2, cm) were measured at the end of the growing season (September 10). Net growth increments were calculated as height increment ($\:\mathrm{H}\mathrm{S}\:=\:\mathrm{H}2-\mathrm{H}1$) and base diameter increment ($\:\mathrm{D}\mathrm{S}\:=\:\mathrm{D}2-\mathrm{D}1$).

To evaluate drought tolerance capacity while mitigating inherent variation among hybrid progenies, the low-water tolerance coefficient (LWindex) was calculated for each trait following the final measurements at the end of the growing season (September 10) [13].

The LWindex was calculated as follows:

$$\begin{aligned} &\mathrm{low}-\mathrm{water}\;\mathrm{tolerance}\;\mathrm{coefficient}\;\left(\mathrm{LWindex},\;\%\right)\;\\&=\;\left(\mathrm{LW}\;\mathrm{trait}\;\mathrm{measurement}\;\mathrm{value}\; \right. \\& \left. \quad /\;\mathrm{NW}\;\mathrm{trait}\;\mathrm{measurement}\;\mathrm{value}\right)\;\times\;100 \end{aligned}$$

### Statistical analysis

#### Statistical software

All analyses were conducted in R (version 4.5.1) [40] using RStudio. The following key R packages were employed: ‘metan’ (v1.19.0) for multi-environment trial analysis and selection indices [41], ‘ggplot2’ (v3.5.2) for visualization [42], ‘RankAggreg’ (v0.6.6) for weighted rank aggregation [43], and ‘bestNormalize’ (v1.9.1) for data normalization [44]. Specifically, the *gamem_met()* function was used for genotype-by-environment analysis, *gmd()* for variance component estimation, *Smith_Hazel()* for computing the SHI, *fai_blup()* for the FAI-BLUP index, and *mgidi()* for the MGIDI. Spearman’s rank correlation was calculated using *corr_coef()*, and correlation networks were visualized with the ‘corrplot’ package [42].

#### Analysis of mixed-effects models

The function *gamem_met()*, as part of the ‘metan’ package [41], was utilized to calculate the following standard linear mixed model [45]: $\:y=X\beta\:+Z\mu\:+\epsilon$. In the model, $\:y$ presented the vector of response variables, $\:\beta\:$ denoted the vector of fixed effects, $\:\mu\:$ signified the vector of random effects; $\:X$ consituted the design matrix composed of 0s and 1s that related $\:y$ to $\:\beta\:$, $\:Z$ was the design matrix composed of 0s and 1s that related $\:y$ to $\:\mu\:$, and $\epsilon$ represented the vector of random errors. Estimates of variance components were obtained via the Expectation-Maximization algorithm using the Restricted Maximum Likelihood (REML) method [46]. In order to ascertain the significance of random effects, a Likelihood Ratio Test (LRT) was performed, which was based on a two-tailed chi-square test with one degree of freedom.

#### Estimation of genetic parameters

The methodology of Olivoto and Lucio (2020) was employed to estimate genetic parameters for traits were estimated using a mixed linear model [41]. Eight heritability estimates were obtained: (i) Broad-sense heritability ($\:{h}_{g}^{2}$), calculated as $\:{h}_{g}^{2}=\frac{{\sigma\:}_{g}^{2}}{{\sigma\:}_{g}^{2}+{\sigma\:}_{i}^{2}+{\sigma\:}_{e}^{2}}$, where $\:{\sigma\:}_{g}^{2}$ represents genetic variance, $\:{\sigma\:}_{i}^{2}$ represents genotype-environment interaction variance, $\:{\sigma\:}_{e}^{2}$ represents environmental variance; (ii) GEIr^2^, the coefficient of determination for genotype-environment interaction effect ($\:{r}^{2}$), calculated as $\:{r}_{i}^{2}=\frac{{\sigma\:}_{i}^{2}}{{\sigma\:}_{g}^{2}+{\sigma\:}_{i}^{2}+{\sigma\:}_{e}^{2}}$; (iii) Mean heritability ($\:{h}_{mg}^{2}$), calculated as $\:{h}_{mg}^{2}=\frac{{\sigma\:}_{g}^{2}}{{\sigma\:}_{g}^{2}+{\sigma\:}_{i}^{2}/e+{\sigma\:}_{e}^{2}/\left(eb\right)}$, where $\:e$ and $\:b$ represented the number of environments and the number of blocks, respectively; (iv) Accuracy, the accuracy of selection ($\:{A}_{c}$), calculated as $\:{A}_{c}=\sqrt{{h}_{mg}^{2}}$; (v) Genotype-environment correlation coefficient ($\:{r}_{ge}$), calculated as $\:{r}_{ge}=\frac{{{\sigma\:}_{g}}^{2}}{{{\sigma\:}_{g}}^{2}+{{\sigma\:}_{i}}^{2}}$; (vi) Genotypic coefficient of variation ($\:{CV}_{g}$), calculated as $\:{CV}_{g}=\left(\frac{\sqrt{{{\sigma\:}_{g}}^{2}}}{\mu\:}\right)\times\:100$, where $\:\mu\:$ representsed the grand mean; (vii) Residual coefficient of variation ($\:{CV}_{r}$), calculated as $\:{CV}_{r}=\left(\frac{\sqrt{{{\sigma\:}_{\mathrm{e}}}^{2}}}{\mu\:}\right)\times\:100$. (viii) CV ratio, given by $\:{CV}_{g}/{CV}_{r}$.

#### Smith-Hazel index (SHI)

The Smith-Hazel index was calculated according to the selection index method developed by Smith (1936) and Hazel (1943) [47, 48]. This index enables the selection of individuals with superior performance for specific traits and is considered one of the most efficient methods for multi-trait selection [49]. The index is computed based on economic weights and the phenotypic and genotypic variance-covariance matrices. The calculation is as follows:

$$\:b={P}^{-1}Gw$$

where $\:P$ and $\:G$ represented the phenotypic and genetic covariance matrices, respectively, $\:b$ and $\:w$ represented the index coefficients and the economic weight vector, respectively.

The calculation method for the individual genotypic genetic value $\:\mathrm{I}$ based on traits $\:x$, $\:y$, …, $\:n$ was as follows:

$$\:I={b}_{x}{G}_{x}+{b}_{y}{G}_{y}+\cdots\:+{b}_{n}{G}_{n}$$

where $\:b$ represented the index coefficients of traits $\:x$, $\:y$, …, $\:n$ respectively, and $\:G$ represented the individual genotypic BLUP values of traits $\:x$, $\:y,$ …, $\:n$ respectively.

#### Factor analytic best linear unbiased prediction (FAI-BLUP)

The FAI-BLUP index is a multi-trait index based on factor analysis and ideotype design that was proposed by Rocha et al. [22].A key advantage of this index is its ability to account for the correlational structure of the data and to incorporate the direction of selection specified by the breeder, thereby facilitating the identification of genotypes closest to the ideotype [50]. Following this methodology, principal component and factor analyses were conducted using breeding values to estimate variance components for each principal component and factor. The genotype-ideotype distance was estimated using the following formula and converted into a spatial probability:

$$\:{P}_{ij}=\frac{\frac{1}{{d}_{ij}}}{{\sum\:}_{i=1;j=1}^{i=n;j=m}\frac{1}{{d}_{ij}}}$$

where $\:{P}_{ij}$ was the similarity probability between the $\:{i}^{th}$($\:i=1,\:2,\dots\:,\:n$) genotype and the $\:{j}^{th}$($\:j=1,\:2,\dots\:,\:m$) ideotype; $\:{d}_{ij}$ was the genotype-ideotype distance between the $\:{i}^{th}$ genotype and the $\:{j}^{th}$ ideotype, calculated based on the standardised mean Euclidean distance.

#### Multi-trait genotype-ideotype distance index (MGIDI)

The Multi-trait Genotype-Ideal Genotype Distance Index (MGIDI) is a novel selection index proposed by Olivoto and Nardino following a comparative evaluation of several multi-trait selection methods [51]. Unlike traditional ranking methods that often rely on post-hoc tests, the MGIDI index enables the direct ranking of genotypes based on their simultaneous performance across multiple traits. Furthermore, it effectively circumvents the issue of multicollinearity among traits by incorporating factor analysis into its construction [52, 53].

In accordance with the methodology established by Olivoto et al., the MGIDI for each genotype was calculated using low-water tolerance coefficient (LWindex) and genotypic values (BLUPs). This served as an indicator for evaluating the stability of each genotype:

$$\:{MGIDI}_{i}={\left[{\sum\:}_{j=1}^{f}{\left({\gamma\:}_{ij}-{\gamma\:}_{j}\right)}^{2}\right]}^{1/2}$$

where $\:{\gamma\:}_{ij}$ denoted the score of the $\:{i}^{th}$ genotype in the $\:{j}^{th}$ factor ($\:i=1,\:2,\:\dots\:,\:g$; $\:i=1,\:2\:,\dots\:,\:f$), and $\:{\gamma\:}_{j}$ denoted the $\:{j}^{th}$ score of the ideotype. The genotypes exhibiting the lowest MGIDI values, i.e. those closest to the ideotype (ID), demonstrated the desired values across all the traits studied.

The proportion of the MGIDI ($\:{\omega\:}_{ij}$) of the $\:{i}^{th}$ genotype accounted for by the $\:{j}^{th}$ factor was utilized to represent the strengths and weaknesses of a genotype, and its calculation formula was as follows:

$$\:{w}_{ij}=\frac{\sqrt{{D}_{ij}^{2}}}{{\sum\:}_{j=i}^{f}\sqrt{{D}_{ij}^{2}}}$$

where $\:{D}_{ij}$ is the distance between the $\:{i}^{th}$ genotype and the ideotype of the $\:{j}^{th}$ factor. A low contribution of a factor is indicative of the fact that the traits in this factor are similar to the designed ideotype.

#### Genetic gain

The genetic gain (GG) of the selected genotypes was calculated using the following formula:

$$\:GG\:\%=\left({X}_{S}{-X}_{0}\right)/{X}_{0}\times\:100$$

where $\:GG\:\%$ is defined as the genetic gain of the selected genotypes, $\:{X}_{S}$ is defined as the mean value (breeding values or drought tolerance coefficients) of traits in the selected genotypes, $\:{X}_{0}$ is defined as the mean value (breeding values or drought tolerance coefficients) of traits in all genotypes.

#### Spearman’s rank correlation coefficient

Spearman’s rank correlation coefficient was used to evaluate the strength and direction of associations among different selection indices (e.g., SHI, FAI-BLUP, MGIDI_BLUP, and MGIDI_LWindex). This non-parametric statistic has been demonstrated to be particularly suitable for ordinal data, or in cases where the assumptions of the Pearson correlation coefficient are not met. By evaluating the rank correlations among these indices, redundancies can be identified and each index can be ensured to provide unique information for the selection process. This was of crucial importance in the development of a comprehensive selection strategy that can effectively balance multiple traits, thereby enhancing the efficiency of breeding programs [54]. In accordance with the methodology proposed by Spearman [55], the calculation formula for Spearman’s rank correlation coefficient was as follows:

$$\:\rho\:=1-\frac{6{d}_{i}^{2}}{n\left({n}^{2}-1\right)}$$

where $\:{d}_{i}$ represented the difference in ranks of the two variables in the $\:{i}^{th}$ observation, and $\:n$ was the number of observations.

#### Weighted Rank Aggregation (WRA)

Weighted Rank Aggregation (WRA) was performed using the ‘RankAggreg’ package in R 4.5.1 software, based on Rank Accuracy (RA) [43]. This method integrates multiple ranking lists into a single, consensus ranking by weighting each according to its reliability. In the context of plant breeding, WRA is particularly valuable for resolving discrepancies among different selection indices, such as SHI, FAI-BLUP, MGIDI_BLUP, and MGIDI_LWindex, thereby ensuring a more robust identification of superior genotypes. It enhances decision-making and selection efficiency in multi-trait analyses by assigning higher weights to indices that are less correlated and demonstrably more reliable.

$$\:{w}_{i}=\frac{\left(1-\left|{r}_{i}\right|\right)}{\left(1-\left|{r}_{i}\right|\right)}$$

Where $\:{r}_{i}$ was the mean absolute Spearman’s rank correlation coefficient for each selection index (SHI, FAI-BLUP, MGIDI_BLUP, MGIDI_LWindex); $\:{w}_{i}$ was the weight assigned to each index based on its Rank Accuracy (RA) [56].

## Results

### Combined ANOVA

Joint analysis of variance revealed that genotype (GEN), environment (ENV), and their interaction (G×E) had significant effects (*P* ≤ 0.05) on most of the 16 measured traits spanning growth, leaf, and photosynthetic physiological characteristics. However, the magnitude and pattern of these effects differed substantially among trait categories and families (Table 1).

**Table 1:** Joint analysis of variance for 16 traits in offspring of three families under LW and NW conditions

Family	Source	df	Mean Squares															
			H2	HS	D2	DS	LA	FW	TW	LDW	LMA	RWC	PN	GA	CI	TR	WUE	LS
‘1-XY’×’N139’	ENV	1	169***	229***	86.6**	21.3*	89.2***	32**	14.2*	3.09ns	42.3*	412***	123***	177***	288***	157***	23.1*	288***
	REP(ENV)	4	1.69***	1.03ns	1.25**	2.09*	0.637ns	1.18ns	0.749ns	4.02**	3.39**	0.158ns	0.232ns	0.705ns	0.0786ns	1.59ns	2.22*	0.0699ns
	GEN	107	3***	0.781ns	3.35***	1.32ns	2.53***	0.855ns	0.921ns	0.996ns	1.27ns	0.274ns	0.552ns	0.795ns	0.561ns	0.68ns	1.03ns	0.559ns
	G×E	107	0.641***	0.712ns	0.698***	1.28***	1.23***	1.04ns	0.999ns	0.948ns	1.12**	0.353ns	0.727ns	0.779ns	0.544ns	0.878ns	1.08ns	0.541ns
	Residuals	428	0.19	0.582	0.285	0.793	0.357	0.952	0.991	0.98	0.785	0.388	0.902	0.698	0.561	0.732	0.909	0.56
‘1-XY’×’N188’	ENV	1	205***	396***	62.6***	3.37ns	394***	9.68*	29.8**	1.23ns	172***	454***	139***	219***	340***	168***	32.3**	340***
	REP(ENV)	4	0.204ns	0.799*	0.468ns	0.941ns	0.138ns	1.17ns	1.09ns	1.15ns	0.682ns	0.404ns	0.13ns	0.741ns	2.33**	0.745ns	0.881ns	2.3**
	GEN	118	2.72***	0.622ns	3.07***	1.37ns	0.516ns	0.958ns	0.98ns	0.966ns	0.837ns	0.354ns	0.985ns	0.684ns	0.524ns	0.699ns	1.13*	0.522ns
	G×E	118	0.819***	0.683***	0.984***	1.72***	0.712***	0.989ns	0.914ns	1.21*	0.846ns	0.354ns	0.844ns	0.519ns	0.42ns	0.797ns	0.789ns	0.42ns
	Residuals	472	0.19	0.324	0.361	0.723	0.367	0.991	0.963	0.951	0.719	0.364	0.758	0.738	0.534	0.764	0.956	0.534
‘ZL-3’×’N188’	ENV	1	134***	61.2ns	65.1***	0.0722ns	496***	1.27ns	61.9**	12.8*	190***	553***	163***	243***	395***	205***	41.2***	395***
	REP(ENV)	4	0.94**	12.2***	0.169ns	0.237ns	0.0782ns	1.47ns	2.93**	0.774ns	0.455ns	0.317ns	1.23ns	0.168ns	1.03ns	0.25ns	0.42ns	1.05ns
	GEN	144	2.74***	1.27ns	3.03***	1.8***	0.502ns	1.16ns	1.08ns	1.05ns	0.818ns	0.358ns	1.02ns	0.672ns	0.56ns	0.985*	1.16ns	0.558ns
	G×E	144	1.21***	1.33***	0.821***	0.943ns	0.512**	0.964ns	0.976ns	1.01ns	0.937*	0.397ns	0.85ns	0.687ns	0.574ns	0.744ns	0.923ns	0.575ns
	Residuals	576	0.282	0.666	0.432	0.82	0.393	0.963	0.866	0.963	0.736	0.352	0.747	0.746	0.532	0.703	0.913	0.532
Joint family	ENV	1	447***	647**	207***	0.979ns	859***	29.6*	99.8**	14.5*	372***	1420***	429***	642***	1020***	532***	89.4***	1020***
	REP(ENV)	4	1.87***	9.48***	0.958*	1.34ns	0.19ns	1.77ns	2.17ns	1.31ns	1.06ns	0.0627ns	0.363ns	0.46ns	0.325ns	0.46ns	0.964ns	0.315ns
	GEN	371	2.98***	1.22*	3.11***	1.72**	1.28**	1.02ns	1.01ns	0.998ns	1.05ns	1.16***	0.874ns	0.707ns	0.548ns	0.801ns	1.11*	0.546ns
	G×E	371	0.919***	1***	0.835***	1.25***	0.983***	1.02ns	0.975ns	1.06ns	0.998***	0.228*	0.811ns	0.661ns	0.516ns	0.799ns	0.924ns	0.515ns
	Residuals	1480	0.223	0.483	0.375	0.753	0.357	0.967	0.935	0.973	0.738	0.196	0.791	0.728	0.546	0.735	0.932	0.546

*H2 *Plant height, *HS* Net increase in plant height, *D2 *Diameter, *DS* Net increase in base diameter, *LA* Leaf area, *FW* Leaf fresh Weight, *TW* Turgid weight, *LDW* Leaf dry weight, *LMA* Leaf Mass per Area, *RWC* Relative water content, *PN* Net photosynthesis rate, *GA* Stomatal conductance, *CI* Intercellular *CO*_*2*_ dioxide concentration, *TR* Transpiration, *WUE* Water use efficiency, *LS* Stomatal limitation

*:*P* ≤ 0.05; **:*P* ≤ 0.01; ***:*P* ≤ 0.001; *ns* Not significant

Growth traits were the most consistently influenced by all three factors. While genotypic effects were widespread in the joint family analysis, they were more variable within individual families. Environment and G×E interactions also showed variable significance across specific families for traits like HS and DS.

Leaf traits exhibited a distinct pattern characterized by the frequent absence of significant G×E interactions. Environmental effects were predominant for most leaf traits, whereas genotypic effects were limited, reaching significance only for LA in one family and RWC in the joint analysis.

Photosynthetic physiological traits demonstrated the most uniform response: environmental effects were universally significant, while G×E interactions were consistently non-significant across all families. Genotypic influence was minimal, with only WUE and TR showing significant effects in specific or joint family analyses.

### Genetic parameters

Genetic parameter estimates for the 16 traits across all families are summarized in Table 2. Broad-sense heritability ($\:{h}_{g}^{2}$) was markedly higher for growth traits than for leaf or photosynthetic physiological traits. H2 consistently showed the highest $\:{h}_{g}^{2}$ across all families (0.301–0.536). Moderate $\:{h}_{g}^{2}$ was observed for a few other traits, such as D2 and LA in specific families, while most traits exhibited low heritability ($\:{h}_{g}^{2}$ < 0.1).

**Table 2:** Genetic parameters for 16 traits in the offspring of three families under LW and NW conditions

Family	Geneticparameters	H2	HS	D2	DS	LA	FW	TW	LDW	LMA	RWC	PN	GA	CI	TR	WUE	LS
‘1-XY’×’N139’	Phenotypic variance	0.734	0.637	0.865	0.962	0.865	0.95	0.981	0.977	0.92	0.363	0.814	0.728	0.558	0.748	0.957	0.557
	Heritability	0.536	0.0181	0.511	0.00734	0.252	0.00	0.00	0.00388	0.0278	0.00	0.00	0.00372	0.000908	0.00	0.00	0.000737
	GEIr^2^	0.205	0.0684	0.159	0.168	0.335	0.00	0.00	0.00	0.12	3.85E-16	0.00	0.0369	0.00	0.021	0.0507	0.00
	h^2^mg	0.786	0.0883	0.792	0.0321	0.516	0.00	0.00	0.0229	0.121	0.00	0.00	0.0204	0.00542	0.00	0.00	0.00441
	Accuracy	0.887	0.297	0.89	0.179	0.718	0.00	0.00	0.151	0.348	0.00	0.00	0.143	0.0736	0.00	0.00	0.0664
	rge	0.441	0.0696	0.325	0.169	0.448	0.00	0.00	0.00	0.123	3.85E-16	0.00	0.0371	0.00	0.021	0.0507	0.00
	CVg	17.3	3.06	17.2	2.50	13.2	0.00	0.00	1.48	3.84	0.00	0.00	1.25	0.54	0.00	0.00	0.512
	CVr	12	21.8	13.8	26.5	16.9	23.4	23.8	23.7	21.3	15.2	22.9	20.1	17.9	20.5	22.9	18.8
	CV ratio	1.44	0.141	1.24	0.0944	0.782	0.00	0.00	0.0624	0.181	0.00	0.00	0.0623	0.0301	0.00	0.00	0.0272
‘1-XY’×’N188’	Phenotypic variance	0.716	0.433	0.916	0.997	0.45	0.985	0.958	0.997	0.76	0.36	0.81	0.693	0.514	0.759	0.957	0.513
	Heritability	0.442	0.00	0.379	0.00	0.00	0.00	0.00471	0.00	0.00	0.00	0.0289	0.00	0.00407	0.00	0.0357	0.00342
	GEIr^2^	0.293	0.253	0.227	0.275	0.183	0.00	0.00	0.0461	0.0535	0.00	0.0353	0.00	2.74E-09	0.00	0.00	0.00
	h^2^mg	0.699	0.00	0.679	0.00	0.00	0.00	0.0276	0.00	0.00	0.00	0.143	0.00	0.0239	0.00	0.182	0.0202
	Accuracy	0.836	0.00	0.824	0.00	0.00	0.00	0.166	0.00	0.00	0.00	0.378	0.00	0.155	0.00	0.426	0.142
	rge	0.525	0.253	0.365	0.275	0.183	0.00	0.00	0.0461	0.0535	0.00	0.0363	0.00	2.75E-09	0.00	0.00	0.00
	CVg	14.4	0.00	14.8	0.00	0.00	0.00	1.60	0.00	0.00	0.00	4.00	0.00	1.09	0.00	4.02	0.999
	CVr	11.2	16.1	15	24.3	14.5	23.7	23.3	23.2	20.2	14.3	22.8	19.8	17	20.8	20.9	17
	CV ratio	1.29	0.00	0.981	0.00	0.00	0.00	0.0688	0.00	0.00	0.00	0.176	0.00	0.0639	0.00	0.192	0.0586
‘ZL-3’×’N188’	Phenotypic variance	0.846	0.877	0.93	1.00	0.431	0.996	0.919	0.985	0.783	0.36	0.809	0.724	0.543	0.757	0.956	0.543
	Heritability	0.301	0.00	0.396	0.142	0.00	0.0334	0.0183	0.00699	0.00	0.00	0.0347	0.00	0.00	0.0531	0.0412	0.00
	GEIr^2^	0.365	0.241	0.14	0.0409	0.0886	5.55E-09	0.0399	0.015	0.0604	0.024	0.0422	0.00	0.0215	0.0181	0.00356	0.0214
	h^2^mg	0.559	0.00	0.729	0.476	0.00	0.172	0.0936	0.0394	0.00	0.00	0.166	0.00	0.00	0.245	0.204	0.00
	Accuracy	0.747	0.00	0.854	0.69	0.00	0.414	0.306	0.198	0.00	0.00	0.407	0.00	0.00	0.495	0.451	0.00
	rge	0.523	0.241	0.231	0.0477	0.0886	5.75E-09	0.0407	0.0151	0.0604	0.024	0.0438	0.00	0.0215	0.0191	0.00371	0.0214
	CVg	13.1	0.00	15.6	8.89	0.00	4.29	3.05	1.95	0.00	0.00	3.94	0.00	0.00	4.72	4.67	0.00
	CVr	13.7	19.2	16.9	21.3	18.5	23.1	21.9	23.1	20.2	15.1	20.3	20	17.2	19.7	22.5	17.2
	CV ratio	0.95	0.00	0.923	0.418	0.00	0.186	0.139	0.0845	0.00	0.00	0.194	0.00	0.00	0.239	0.208	0.00
Joint family	Phenotypic variance	0.798	0.692	0.908	0.998	0.616	0.985	0.953	0.992	0.833	0.363	0.808	0.713	0.542	0.757	0.96	0.541
	Heritability	0.43	0.0525	0.418	0.0782	0.0811	0.000492	0.00536	0.00	0.0103	0.43	0.0129	0.00	0.00228	0.000498	0.0307	0.00173
	GEIr^2^	0.29	0.25	0.169	0.167	0.339	0.0178	0.0138	0.019	0.104	0.0286	0.00849	0.00	0.00	0.0282	1.39E-09	0.00
	h^2^mg	0.691	0.179	0.732	0.272	0.234	0.00284	0.0305	0.00	0.049	0.805	0.0717	0.00	0.0135	0.00282	0.16	0.0103
	Accuracy	0.831	0.423	0.855	0.521	0.483	0.0533	0.175	0.00	0.221	0.897	0.268	0.00	0.116	0.0531	0.4	0.102
	rge	0.509	0.264	0.29	0.181	0.369	0.0178	0.0139	0.019	0.105	0.0503	0.0086	0.00	0.00	0.0282	1.43E-09	0.00
	CVg	13.1	4.87	16	6.98	4.96	0.488	1.58	0.00	2.05	9.14	2.27	0.00	0.779	0.431	3.81	0.708
	CVr	10.6	17.8	15.9	21.7	13.2	21.8	21.4	22.8	19	10.3	19.7	18.7	16.3	19	21.4	17
	CV ratio	1.24	0.274	1.01	0.322	0.374	0.0224	0.0739	0.00	0.108	0.892	0.115	0.00	0.0478	0.0227	0.178	0.0417

*H2 *Plant height, *HS* Net increase in plant height, *D2 *Diameter, *DS* Net increase in base diameter, *LA* Leaf area, *FW* Leaf fresh Weight, *TW* Turgid weight, *LDW* Leaf dry weight, *LMA* Leaf Mass per Area, *RWC* Relative water content, *PN* Net photosynthesis rate, *GA* Stomatal conductance, *CI* Intercellular *CO*_*2*_ dioxide concentration, *TR* Transpiration, *WUE* Water use efficiency, *LS* Stomatal limitation

*:*P* ≤ 0.05; **:*P* ≤ 0.01; ***:*P* ≤ 0.001; *ns* Not significant

Mean-based heritability ($\:{h}_{mg}^{2}$), indicative of selection efficiency at the genotype mean level, followed a similar pattern but with higher absolute values. Notably, $\:{h}_{mg}^{2}$ exceeded 0.5 consistently only for the key growth traits H2 and D2 across all families.

The genotype-by-environment interaction (G×E) was minimal for the vast majority of traits, as evidenced by low or zero genotype-by-environment interaction determination coefficients (GEIr^2^). A substantial GEIr^2^ was observed only for H2 in the ‘ZL-3’×’N188’ family.

Accordingly, selection accuracy and genotype–environment correlation ($\:{r}_{ge}$) were generally high only for the main growth traits. Selection accuracy exceeded 0.5 for H2 and D2 in all families, and $\:{r}_{ge}$ was > 0.5 for H2 in most family and joint analyses. For most other traits, both parameters were low, reflecting a larger environmental influence on their phenotypic expression.

Finally, the ratio of genotypic to residual coefficient of variation (CV ratio) was greater than 1 only for H2 and D2 in specific families, underscoring the greater genetic relative to non-genetic variability for these core growth traits.

### SHI-based selection of hybrid progeny genotypes

**Fig. 1:**
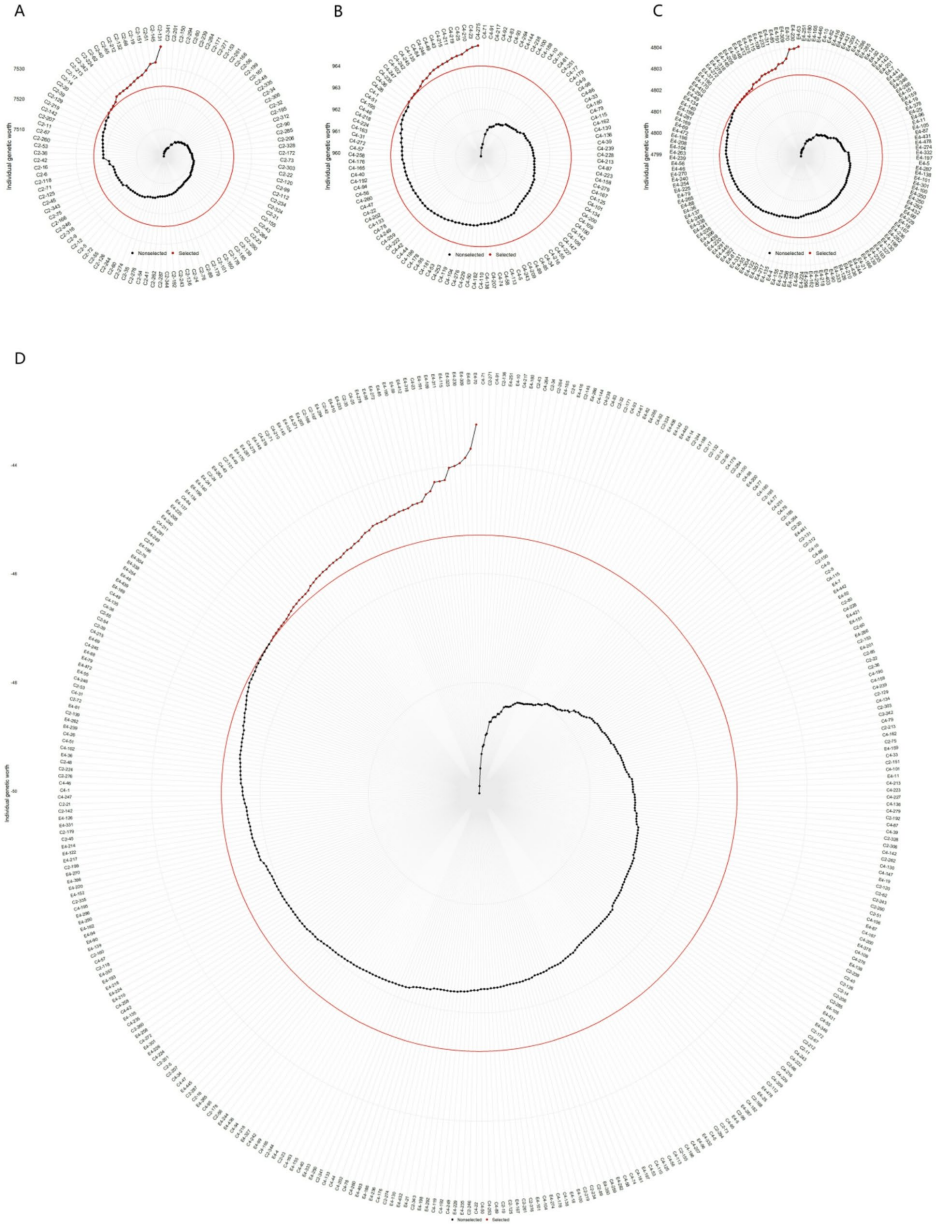
Genotype ranking and selection based on the Smith-Hazel index for (**A**) family C2, ‘1-XY’×’N139’, (**B**) family C4, ‘1-XY’×’N188’, (**C**) family E4, ‘ZL-3’×’N188’, and (**D**) the joint family. Selected genotypes are highlighted in red, with a red circle indicating the selection threshold

The Smith-Hazel index (SHI) was used to select genotypes with superior multi-trait performance (Table 3; Fig. 1). Applying a 20% selection intensity identified 16, 18, 22, and 56 top-ranked genotypes from the ‘1‑XY’×’N139’, ‘1‑XY’×’N188’, ‘ZL‑3’×’N188’, and joint families, respectively (Fig. 1A-D). Representative selections included genotypes such as C2‑131, C4‑275, E4‑63, and E4‑70.

**Table 3:** Selection differential and index coefficient of the selected clones using SHI

‘1-XY’×’N139’								
**Traits**	**Xo**	**Xs**	**SD**	**SD%**	**h2**	**SG**	**SG%**	**Index coefficient**
H2	3.63	3.4	-0.224	-6.16	0.667	-0.149	-4.11	0.094
HS	3.5	3.5	0	0	0	0	0	-0.953
D2	3.87	3.57	-0.3	-7.75	0.728	-0.218	-5.64	3.05
DS	3.36	3.34	-0.0293	-0.871	0.175	-0.00513	-0.152	-3.29
LA	3.54	2.84	-0.705	-19.9	0.569	-0.401	-11.3	-22.8
FW	4.17	4.17	0	0	0	0	0	-2.36
TW	4.17	4.17	0	0	0	0	0	0.951
LDW	4.17	4.16	-0.00352	-0.0845	0.00804	-0.0000283	-0.000679	30.1
LMA	4.17	4.26	0.093	2.23	0.153	0.0142	0.341	-42.3
RWC	3.96	3.96	0	0	0	0	0	5.78
PN	3.95	3.95	0	0	0	0	0	-18.4
GA	4.17	4.17	0	0	0	0	0	-0.153
CI	4.17	4.17	0	0	0	0	0	930
TR	4.17	4.17	0	0	0	0	0	19.8
WUE	4.17	4.17	0.000141	0.00339	0.0195	0.00000275	0.0000661	15
LS	3.96	3.96	0	0	0	0	0	931

‘1-XY’×’N188’								
H2	3.9	4.46	0.553	14.2	0.608	0.336	8.62	0.39
HS	3.54	3.54	0	0	0	0	0	-0.102
D2	3.99	4.77	0.78	19.5	0.677	0.528	13.2	1.41
DS	3.5	3.59	0.0931	2.66	0.193	0.0179	0.513	0.242
LA	4.19	4.19	0	0	0	0	0	0.669
FW	4.19	4.19	0	0	0	0	0	0.684
TW	4.19	4.19	0	0	0	0	0	-0.658
LDW	4.19	4.19	0	0	0	0	0	-1.34
LMA	4.19	4.19	0	0	0	0	0	1.15
RWC	4.19	4.19	0	0	0	0	0	0.0939
PN	3.82	3.82	0	0	0	0	0	0.238
GA	4.19	4.19	0	0	0	0	0	-0.229
CI	4.19	4.19	0	0	0	0	0	113
TR	4.19	4.19	0	0	0	0	0	0.33
WUE	4.59	4.61	0.0183	0.398	0.0718	0.00131	0.0286	0.178
LS	4.19	4.19	0	0	0	0	0	114

‘ZL-3’×’N188’								
H2	3.87	4.45	0.588	15.2	0.615	0.362	9.36	0.556
HS	4.25	4.33	0.0752	1.77	0.166	0.0125	0.293	-0.0764
D2	3.9	4.58	0.677	17.4	0.67	0.453	11.6	1.22
DS	4.25	4.57	0.314	7.4	0.363	0.114	2.68	0.711
LA	3.38	3.38	0	0	0	0	0	-0.0106
FW	4.25	4.22	-0.0342	-0.806	0.0936	-0.0032	-0.0754	1.35
TW	4.25	4.23	-0.0163	-0.384	0.0488	-0.000797	-0.0188	-2.12
LDW	4.25	4.25	0.00281	0.066	0.03	0.0000841	0.00198	-2.25
LMA	4.25	4.25	0	0	0	0	0	0.908
RWC	3.92	3.92	0	0	0	0	0	-0.238
PN	4.25	4.25	0.0000838	0.00197	0.0136	0.00000114	0.0000268	0.441
GA	4.25	4.25	0	0	0	0	0	-1.78
CI	4.25	4.25	0	0	0	0	0	566
TR	4.25	4.25	0	0	0	0	0	-0.298
WUE	4.25	4.24	-0.00817	-0.192	0.0892	-0.000729	-0.0171	-1.24
LS	4.25	4.25	0	0	0	0	0	566

Joint family								
H2	4.45	5.17	0.719	16.1	0.663	0.476	10.7	0.971
HS	3.91	3.98	0.0679	1.74	0.13	0.00881	0.225	0.127
D2	3.86	4.57	0.706	18.3	0.687	0.485	12.6	1.04
DS	4	4.23	0.231	5.77	0.338	0.0779	1.95	0.324
LA	4.51	4.53	0.0239	0.531	0.152	0.00364	0.0807	-1.37
FW	4.51	4.51	-0.00189	-0.042	0.015	-0.0000284	-0.000629	0.339
TW	4.51	4.51	-0.000826	-0.0183	0.00497	-0.00000411	-0.0000911	-0.486
LDW	4.32	4.32	-0.0000382	-0.000884	0.000705	-2.69E-08	-0.000000623	2.2
LMA	4.51	4.51	-0.00433	-0.096	0.0294	-0.000127	-0.00282	-3.23
RWC	4.32	4.35	0.0269	0.622	0.0928	0.00249	0.0577	1.36
PN	4.51	4.51	0	0	0	0	0	0.367
GA	4.51	4.51	0	0	0	0	0	-0.72
CI	4.51	4.51	0	0	0	0	0	-4.9
TR	4.51	4.51	0	0	0	0	0	-0.0823
WUE	4.51	4.51	-0.00461	-0.102	0.0621	-0.000286	-0.00634	-0.603
LS	4.32	4.32	0	0	0	0	0	-5.5

*H2 *Plant height, *HS* Net increase in plant height, *D2 *Diameter, *DS* Net increase in base diameter, *LA* Leaf area, *FW* Leaf fresh Weight, *TW* Turgid weight; LDW: Leaf dry weight, *LMA* Leaf Mass per Area, *RWC* Relative water content, *PN* Net photosynthesis rate, *GA* Stomatal conductance, *CI* Intercellular *CO*_*2*_ dioxide concentration, *TR* Transpiration, *WUE* Water use efficiency, *LS* Stomatal limitation

The SHI exhibited a strong and consistent directional selection for growth traits across families, with H2 and D2 receiving the strongest positive selection pressure. In contrast, selection for most leaf and photosynthetic physiological traits was weak or neutral, as indicated by near-zero selection differentials (SD) and genetic gains (SG) for traits such as LA, LDW, LMA, and HS in multiple families.

Accordingly, SHI-based genetic gains were highest for the targeted growth traits (Table 3). D2 showed the most substantial and consistent improvement across all selection scenarios (SG% up to 12.6%), followed by H2 (SG% up to 10.7%). Gains for other traits were minimal or, in the case of LA in one family, negative (–11.3%). Notably, genetic gains for key growth traits were generally higher in the joint family analysis than within individual families, highlighting the benefit of pooled selection across a broader genetic base.

### Selection of hybrid progeny genotypes based on FAI-BLUP

Factor analysis based on the FAI-BLUP index retained seven factors, collectively explaining 79.2–82.2% of the total phenotypic variance across families (Table 4). While the factor solutions shared a consistent dimensionality, the patterns of trait association within factors (e.g., the grouping of specific growth, leaf, and photosynthetic physiological traits) varied among families, reflecting their distinct genetic backgrounds.

**Table 4:** EFA overlay retained factor eigenvalues, variances, loadings, and FAI_BLUP selection gains

‘1-XY’×’N139’													
**Traits**	**FA1**	**FA2**	**FA3**	**FA4**	**FA5**	**FA6**	**FA7**	**Comunality**	**Factor**	**Xo**	**Xs**	**SD**	**SD%**
H2	-0.752	0.127	0.0218	-0.158	-0.226	-0.014	0.25	0.721	FA1	3.63	3.75	0.118	3.26
HS	-0.233	0.207	0.0381	0.111	-0.0654	0.0218	0.607	0.485	FA7	3.5	3.56	0.0535	1.53
D2	-0.909	0.0382	0.0645	-0.0999	-0.177	0.021	0.0742	0.878	FA1	3.87	4.17	0.303	7.84
DS	-0.817	0.00421	-0.0174	0.101	0.101	-0.0261	0.05	0.691	FA1	3.36	3.76	0.393	11.7
LA	-0.436	-0.211	0.0785	-0.118	-0.632	0.251	-0.281	0.796	FA5	3.54	3.34	-0.201	-5.68
FW	-0.0234	-0.0237	0.976	-0.0397	0.106	-0.002	-0.0646	0.97	FA3	4.17	4.19	0.024	0.576
TW	-0.0354	-0.0187	0.975	-0.00719	-0.0278	-0.0709	0.0532	0.962	FA3	4.17	4.12	-0.0461	-1.11
LDW	-0.193	-0.308	0.144	-0.161	0.681	0.22	-0.301	0.782	FA5	4.17	4.45	0.281	6.74
LMA	0.206	-0.053	0.0406	-0.0219	0.964	-0.0741	-0.0183	0.983	FA5	4.17	4.49	0.32	7.67
RWC	0.0558	0.308	-0.00172	-0.0303	0.00204	-0.0259	-0.615	0.478	FA7	3.96	3.88	-0.0796	-2.01
PN	0.0229	0.117	-0.0691	-0.0346	-0.0438	0.952	0.0923	0.938	FA6	3.95	3.98	0.0312	0.789
GA	-0.0489	-0.346	-0.122	-0.367	0.025	0.168	0.474	0.525	FA7	4.17	4.14	-0.0294	-0.705
CI	0.0684	-0.951	0.029	0.0646	0.0566	-0.0731	0.0625	0.927	FA2	4.17	4.27	0.0986	2.37
TR	-0.0417	0.0907	-0.00524	-0.857	0.00239	0.417	-0.0163	0.919	FA4	4.17	3.97	-0.194	-4.65
WUE	0.0627	-0.0756	-0.0798	0.933	-0.0512	0.257	0.0807	0.961	FA4	4.17	4.46	0.295	7.07
LS	-0.0715	0.951	-0.0272	-0.0665	-0.0532	0.0737	-0.0618	0.926	FA2	3.96	3.86	-0.0965	-2.44
Eigenvalues	3.06	2.33	2.08	1.88	1.37	1.16	1.07						
Variance (%)	19.1	14.6	13.0	11.7	8.6	7.2	6.7						
Cum. variance (%)	19.1	33.7	46.7	58.4	67	74.2	80.9						

‘1-XY’×’N188’													
H2	-0.122	-0.852	-0.16	-0.0347	-0.126	0.0221	-0.0397	0.785	FA2	3.9	4.13	0.229	5.86
HS	0.0542	0.0476	-0.117	-0.0765	-0.166	-0.00522	0.879	0.825	FA7	3.54	3.6	0.0655	1.85
D2	-0.0369	-0.932	-0.06	-0.0351	0.138	0.0495	0.0278	0.898	FA2	3.99	4.28	0.286	7.17
DS	0.224	-0.6	0.293	0.0941	0.262	0.105	-0.0679	0.589	FA2	3.5	3.49	-0.0133	-0.38
LA	0.116	-0.0336	0.246	0.569	0.0487	-0.118	0.415	0.588	FA4	4.19	4.09	-0.107	-2.54
FW	-0.00554	0.0495	0.941	-0.171	-0.0814	0.0532	-0.0209	0.926	FA3	4.19	4.12	-0.074	-1.76
TW	-0.0139	0.0293	0.945	0.0249	-0.129	0.012	-0.0473	0.914	FA3	4.19	4.07	-0.128	-3.06
LDW	0.00845	0.0000579	0.208	-0.844	0.0739	-0.122	0.208	0.82	FA4	4.19	4.47	0.272	6.49
LMA	-0.0717	-0.0316	0.0613	-0.982	0.0261	-0.0588	-0.0141	0.979	FA4	4.19	4.48	0.284	6.77
RWC	0.0282	-0.000534	-0.106	-0.0832	0.383	0.0851	-0.12	0.187	FA5	4.19	4.3	0.107	2.54
PN	0.0679	0.0704	-0.0573	-0.0627	0.107	-0.974	0.0394	0.978	FA6	3.82	4	0.179	4.68
GA	-0.58	-0.15	-0.127	-0.14	-0.179	-0.0415	-0.171	0.457	FA1	4.19	4.32	0.124	2.96
CI	-0.967	0.0461	0.0593	-0.00257	0.0677	0.0736	0.0226	0.951	FA1	4.19	4.41	0.214	5.11
TR	0.0713	0.138	-0.0131	-0.0942	-0.651	-0.696	-0.0148	0.941	FA6	4.19	4.05	-0.139	-3.32
WUE	-0.0148	-0.106	-0.052	0.0283	0.907	-0.208	0.00365	0.881	FA5	4.59	4.99	0.402	8.76
LS	0.967	-0.0467	-0.0585	0.000928	-0.0676	-0.0739	-0.0215	0.951	FA1	4.19	3.98	-0.214	-5.11
Eigenvalues	2.5	2.44	2.08	1.99	1.43	1.23	1						
Variance (%)	15.6	15.3	13	12.4	8.9	7.7	6.3						
Cum. variance (%)	15.6	30.9	43.9	56.3	65.2	72.9	79.2						

‘ZL-3’×’N188’													
H2	-0.154	-0.837	0.0395	-0.0296	-0.0237	0.0209	-0.0616	0.731	FA2	3.87	3.84	-0.0288	-0.745
HS	0.0427	-0.431	0.0597	-0.152	-0.0301	-0.183	-0.331	0.358	FA2	4.25	4.21	-0.0421	-0.991
D2	-0.109	-0.913	-0.0281	0.012	-0.035	0.0376	0.0509	0.852	FA2	3.9	4.02	0.125	3.2
DS	-0.0275	-0.793	-0.0297	-0.0139	0.00173	-0.0276	0.143	0.652	FA2	4.25	4.35	0.0991	2.33
LA	-0.155	-0.0649	0.137	-0.248	0.173	-0.723	-0.245	0.721	FA6	3.38	3.31	-0.0677	-2
FW	0.956	0.137	-0.162	-0.06	0.0589	-0.0442	-0.0304	0.969	FA1	4.25	4.68	0.43	10.1
TW	0.965	0.118	-0.0168	-0.0985	0.0619	0.0452	-0.0395	0.963	FA1	4.25	4.6	0.354	8.33
LDW	0.0522	-0.0347	-0.958	-0.103	0.118	-0.118	-0.0392	0.962	FA3	4.25	4.5	0.249	5.85
LMA	0.125	0.0491	-0.954	0.033	0.0556	0.2	0.0866	0.98	FA3	4.25	4.5	0.247	5.81
RWC	0.135	0.0164	-0.0507	0.281	-0.146	-0.727	0.211	0.695	FA6	3.92	4.07	0.146	3.71
PN	-0.0611	-0.0625	-0.00961	-0.0419	0.0972	-0.0225	0.901	0.832	FA7	4.25	4.37	0.119	2.81
GA	-0.166	0.124	0.19	-0.252	-0.59	-0.00248	0.251	0.554	FA5	4.25	4.19	-0.0567	-1.33
CI	-0.0112	-0.0812	0.0327	0.138	-0.943	0.00354	-0.15	0.939	FA5	4.25	4.34	0.0892	2.1
TR	0.0398	-0.096	-0.0515	-0.725	0.111	-0.0172	0.632	0.952	FA4	4.25	4.22	-0.031	-0.729
WUE	-0.144	0.0531	0.0553	0.908	-0.0535	-0.0366	0.0519	0.857	FA4	4.25	4.43	0.177	4.17
LS	0.0121	0.0822	-0.0346	-0.138	0.943	-0.00444	0.151	0.939	FA5	4.25	4.16	-0.0893	-2.1
Eigenvalues	3.02	2.55	1.93	1.68	1.58	1.11	1.09						
Variance (%)	18.9	15.9	12.1	10.5	9.9	6.9	6.8						
Cum. variance (%)	18.9	34.8	46.9	57.4	67.3	74.2	81						

Joint family													
H2	-0.833	-0.0517	0.0895	-0.0846	-0.0744	-0.167	0.00977	0.745	FA1	4.45	4.73	0.274	6.16
HS	-0.246	-0.0181	0.0345	-0.057	0.0283	-0.667	0.0845	0.519	FA6	3.91	4.03	0.115	2.93
D2	-0.95	-0.0229	0.0409	0.00768	-0.0282	-0.0124	-0.00977	0.905	FA1	3.86	4.25	0.389	10.1
DS	-0.721	0.0617	-0.0321	0.0652	0.024	-0.311	-0.0081	0.627	FA1	4	4.27	0.264	6.6
LA	-0.152	0.0318	-0.0882	-0.0459	-0.336	-0.667	-0.1	0.601	FA6	4.51	4.5	-0.00614	-0.136
FW	0.0581	0.0373	-0.957	-0.0438	0.157	-0.138	0.0304	0.967	FA3	4.51	4.55	0.0437	0.968
TW	0.0401	0.0222	-0.976	-0.0511	-0.00402	0.117	0.0209	0.972	FA3	4.51	4.48	-0.0335	-0.742
LDW	-0.016	0.00518	-0.114	-0.0548	0.907	-0.0947	-0.0754	0.853	FA5	4.32	4.66	0.341	7.89
LMA	0.0913	-0.0159	-0.0424	-0.011	0.915	0.36	0.0108	0.977	FA5	4.51	4.79	0.278	6.16
RWC	-0.0588	0.0202	0.0133	0.0481	-0.00554	-0.866	0.00814	0.756	FA6	4.32	4.43	0.107	2.47
PN	0.00151	0.0542	0.0505	0.0767	0.0392	0.0205	-0.981	0.976	FA7	4.51	4.6	0.0866	1.92
GA	-0.0244	-0.57	0.143	-0.254	-0.0471	0.0346	-0.156	0.438	FA2	4.51	4.54	0.0335	0.742
CI	0.00238	-0.957	-0.0331	0.13	0.029	-0.00118	0.113	0.947	FA2	4.51	4.7	0.189	4.2
TR	-0.0135	0.0558	-0.0183	-0.726	0.0643	-0.0116	-0.649	0.957	FA4	4.51	4.36	-0.151	-3.35
WUE	0.00766	-0.0318	0.0944	0.965	-0.0371	0.0339	-0.146	0.966	FA4	4.51	4.82	0.309	6.86
LS	-0.00196	0.957	0.0326	-0.13	-0.0282	-0.00011	-0.113	0.947	FA2	4.32	4.13	-0.188	-4.36
Eigenvalues	3.01	2.37	2.04	1.83	1.58	1.19	1.13						
Variance (%)	18.8	14.9	12.7	11.4	9.9	7.5	7						
Cum. variance (%)	18.8	33.7	46.4	57.8	67.7	75.2	82.2						

*H2 *Plant height, *HS *Net increase in plant height, *D2 *Diameter, *DS *Net increase in base diameter, *LA *Leaf area, *FW *Leaf fresh Weight, *TW *Turgid weight, *LDW *Leaf dry weight, *LMA *Leaf Mass per Area, *RWC *Relative water content, *PN *Net photosynthesis rate, *GA *Stomatal conductance, *CI *Intercellular *CO*_*2*_ dioxide concentration, *TR *Transpiration, *WUE *Water use efficiency, *LS *Stomatal limitation

**Fig. 2:**
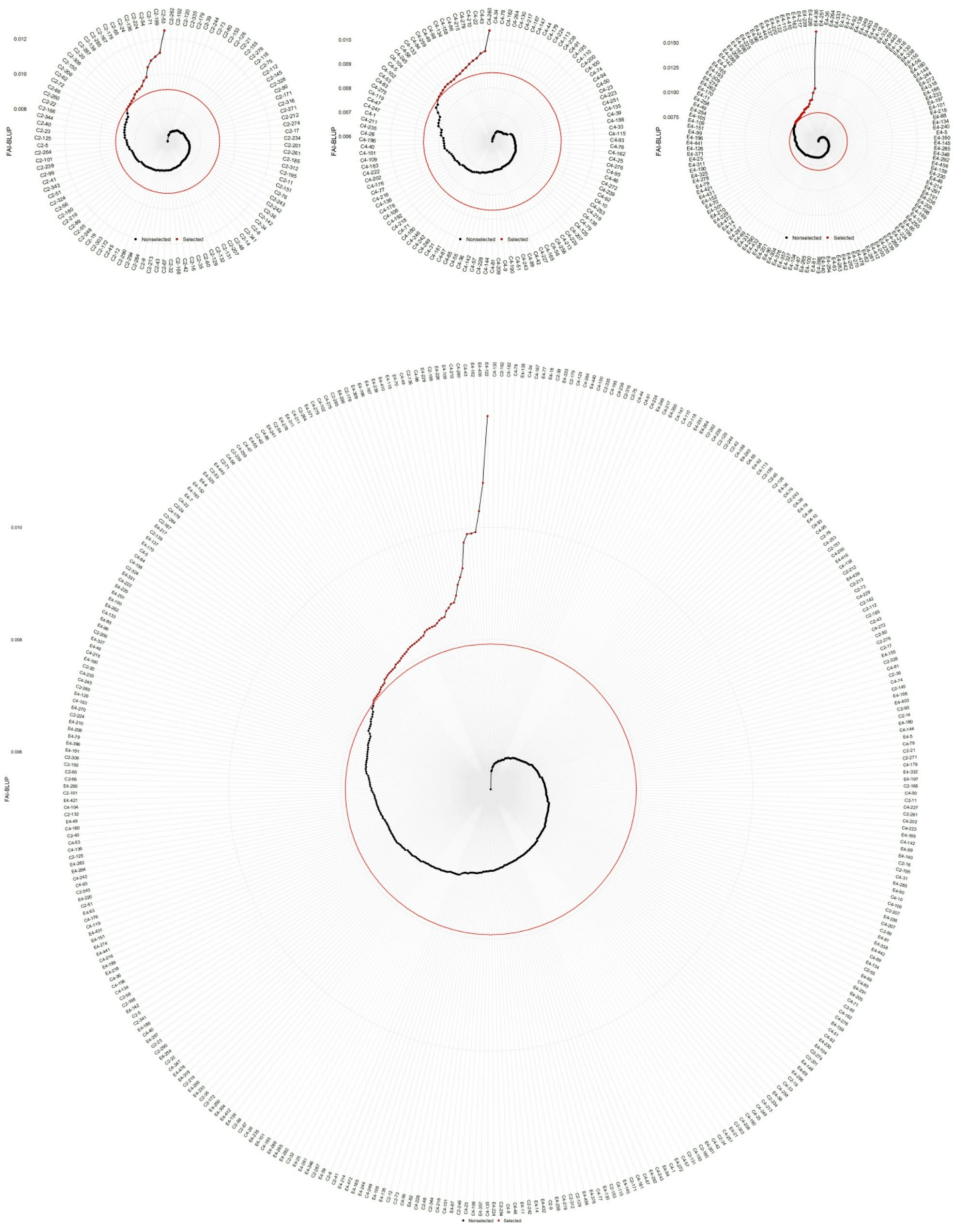
Genotype ranking and selection based on the FAI_BLUP for (**A**) family C2, ‘1-XY’×’N139’, (**B**) family C4, ‘1-XY’×’N188’, (**C**) family E4, ‘ZL-3’×’N188’, and (**D**) the joint family. Selected genotypes are highlighted in red, with a red circle indicating the selection threshold

Using this index with a 20% selection intensity, 16, 18, 22, and 56 top-performing genotypes were selected from the ‘1‑XY’×’N139’, ‘1‑XY’×’N188’, ‘ZL‑3’×’N188’, and joint families, respectively (Fig. 2A-D). Representative genotypes included C2‑53, C4‑260, E4‑436, and E4‑122.

In contrast to the SHI, the FAI-BLUP index facilitated more balanced multi-trait selection. Selection gains (SG%) varied by family but were distributed across a wider range of trait categories (Table 4). For instance, substantial positive gains were observed not only for growth traits like D2 (up to 10.1%) but also for leaf traits such as FW (10.1% in one family) and photosynthetic physiological traits like WUE (8.76% in another). This indicates the index’s capacity to identify genotypes with coordinated improvement across different trait modules, rather than strongly favoring growth traits alone.

### Selection of hybrid progeny genotypes based on MGIDI_BLUP

The MGIDI_BLUP index, derived from factor analysis of genotypic values, similarly retained a stable seven-factor structure across all families, explaining 79.2–82.2% of the total variance (Table 5). This confirms a consistent multi-dimensional framework for trait integration, while the specific loading patterns of growth, leaf, and photosynthetic physiological traits onto factors varied among genetic backgrounds, mirroring the findings from the FAI-BLUP analysis.

**Table 5:** EFA overlay retained factor eigenvalues, variances, loadings, and MGIDI_BLUP selection gains

‘1-XY’×’N139’														
**Traits**	**FA1**	**FA2**	**FA3**	**FA4**	**FA5**	**FA6**	**FA7**	**Communality**	**Uniquenesses**	**Factor**	**Xo**	**Xs**	**SD**	**SD%**
H2	-0.752	0.127	-0.0218	-0.158	0.226	-0.014	0.25	0.721	0.279	FA1	3.63	3.84	0.208	5.75
HS	-0.233	0.207	-0.0381	0.111	0.0654	0.0218	0.607	0.485	0.515	FA7	3.5	3.56	0.0566	1.61
D2	-0.909	0.0382	-0.0645	-0.0999	0.177	0.021	0.0742	0.878	0.122	FA1	3.87	4.22	0.353	9.13
DS	-0.817	0.00421	0.0174	0.101	-0.101	-0.0261	0.05	0.691	0.309	FA1	3.36	3.77	0.408	12.1
LA	0.436	0.211	0.0785	0.118	-0.632	-0.251	0.281	0.796	0.204	FA5	3.54	3.36	-0.186	-5.24
FW	0.0234	0.0237	0.976	0.0397	0.106	0.002	0.0646	0.97	0.0301	FA3	4.17	4.21	0.0456	1.09
TW	-0.0354	-0.0187	-0.975	-0.00719	0.0278	-0.0709	0.0532	0.962	0.0385	FA3	4.17	4.16	-0.00992	-0.238
LDW	-0.193	-0.308	-0.144	-0.161	-0.681	0.22	-0.301	0.782	0.218	FA5	4.17	4.43	0.265	6.36
LMA	0.206	-0.053	-0.0406	-0.0219	-0.964	-0.0741	-0.0183	0.983	0.0168	FA5	4.17	4.47	0.301	7.22
RWC	0.0558	0.308	0.00172	-0.0303	-0.00204	-0.0259	-0.615	0.478	0.522	FA7	3.96	3.88	-0.0753	-1.9
PN	0.0229	0.117	0.0691	-0.0346	0.0438	0.952	0.0923	0.938	0.0625	FA6	3.95	3.96	0.0151	0.383
GA	0.0489	0.346	-0.122	0.367	0.025	-0.168	-0.474	0.525	0.475	FA7	4.17	4.13	-0.0412	-0.988
CI	0.0684	-0.951	-0.029	0.0646	-0.0566	-0.0731	0.0625	0.927	0.0726	FA2	4.17	4.28	0.11	2.63
TR	0.0417	-0.0907	-0.00524	0.857	0.00239	-0.417	0.0163	0.919	0.0809	FA4	4.17	3.96	-0.206	-4.95
WUE	0.0627	-0.0756	0.0798	0.933	0.0512	0.257	0.0807	0.961	0.0394	FA4	4.17	4.46	0.296	7.1
LS	0.0715	-0.951	-0.0272	0.0665	-0.0532	-0.0737	0.0618	0.926	0.0738	FA2	3.96	3.85	-0.107	-2.71
Eigenvalues	3.06	2.33	2.08	1.88	1.37	1.16	1.07							
Variance (%)	19.1	14.5	13	11.7	8.56	7.23	6.69							
Cum. variance (%)	19.1	33.7	46.7	58.4	67	74.2	80.9							

‘1-XY’×’N188’														
H2	-0.122	-0.852	-0.16	0.0347	-0.126	0.0221	0.0397	0.785	0.215	FA2	3.9	4.13	0.231	5.91
HS	0.0542	0.0476	-0.117	0.0765	-0.166	-0.00522	-0.879	0.825	0.175	FA7	3.54	3.83	0.288	8.15
D2	-0.0369	-0.932	-0.06	0.0351	0.138	0.0495	-0.0278	0.898	0.102	FA2	3.99	4.41	0.42	10.5
DS	0.224	-0.6	0.293	-0.0941	0.262	0.105	0.0679	0.589	0.411	FA2	3.5	3.63	0.126	3.6
LA	-0.116	0.0336	-0.246	0.569	-0.0487	0.118	0.415	0.588	0.412	FA4	4.19	4.17	-0.0206	-0.492
FW	0.00554	-0.0495	-0.941	-0.171	0.0814	-0.0532	-0.0209	0.926	0.0737	FA3	4.19	4.25	0.0539	1.28
TW	-0.0139	0.0293	0.945	-0.0249	-0.129	0.012	0.0473	0.914	0.086	FA3	4.19	4.19	-0.00487	-0.116
LDW	0.00845	0.0000579	0.208	0.844	0.0739	-0.122	-0.208	0.82	0.18	FA4	4.19	4.53	0.332	7.92
LMA	-0.0717	-0.0316	0.0613	0.982	0.0261	-0.0588	0.0141	0.979	0.021	FA4	4.19	4.48	0.285	6.8
RWC	0.0282	-0.000534	-0.106	0.0832	0.383	0.0851	0.12	0.187	0.813	FA5	4.19	4.24	0.0459	1.09
PN	0.0679	0.0704	-0.0573	0.0627	0.107	-0.974	-0.0394	0.978	0.0224	FA6	3.82	3.94	0.122	3.19
GA	0.58	0.15	0.127	-0.14	0.179	0.0415	-0.171	0.457	0.543	FA1	4.19	4.23	0.0326	0.776
CI	-0.967	0.0461	0.0593	0.00257	0.0677	0.0736	-0.0226	0.951	0.0492	FA1	4.19	4.23	0.0398	0.949
TR	-0.0713	-0.138	0.0131	-0.0942	0.651	0.696	-0.0148	0.941	0.0595	FA6	4.19	4.09	-0.104	-2.49
WUE	-0.0148	-0.106	-0.052	-0.0283	0.907	-0.208	-0.00365	0.881	0.119	FA5	4.59	4.89	0.297	6.46
LS	-0.967	0.0467	0.0585	0.000928	0.0676	0.0739	-0.0215	0.951	0.0486	FA1	4.19	4.16	-0.0392	-0.935
Eigenvalues	2.5	2.44	2.08	1.99	1.43	1.23	1							
Variance (%)	15.6	15.3	13	12.4	8.93	7.71	6.26							
Cum. variance (%)	15.6	30.9	43.9	56.3	65.2	72.9	79.2							

‘ZL-3’×’N188’														
H2	-0.154	0.837	-0.0395	-0.0296	-0.0237	0.0209	-0.0616	0.731	0.269	FA2	3.87	4.18	0.318	8.24
HS	0.0427	0.431	-0.0597	-0.152	-0.0301	-0.183	-0.331	0.358	0.642	FA2	4.25	4.33	0.0833	1.96
D2	-0.109	0.913	0.0281	0.012	-0.035	0.0376	0.0509	0.852	0.148	FA2	3.9	4.31	0.415	10.6
DS	-0.0275	0.793	0.0297	-0.0139	0.00173	-0.0276	0.143	0.652	0.348	FA2	4.25	4.5	0.251	5.91
LA	0.155	-0.0649	0.137	0.248	-0.173	0.723	0.245	0.721	0.279	FA6	3.38	3.28	-0.098	-2.9
FW	-0.956	0.137	-0.162	0.06	-0.0589	0.0442	0.0304	0.969	0.0311	FA1	4.25	4.35	0.103	2.43
TW	0.965	-0.118	0.0168	-0.0985	0.0619	0.0452	-0.0395	0.963	0.0367	FA1	4.25	4.32	0.0644	1.51
LDW	0.0522	0.0347	0.958	-0.103	0.118	-0.118	-0.0392	0.962	0.0378	FA3	4.25	4.4	0.144	3.39
LMA	0.125	-0.0491	0.954	0.033	0.0556	0.2	0.0866	0.98	0.0198	FA3	4.25	4.42	0.169	3.97
RWC	0.135	-0.0164	0.0507	0.281	-0.146	-0.727	0.211	0.695	0.305	FA6	3.92	4.02	0.1	2.55
PN	-0.0611	0.0625	0.00961	-0.0419	0.0972	-0.0225	0.901	0.832	0.168	FA7	4.25	4.32	0.0721	1.7
GA	0.166	0.124	0.19	0.252	0.59	0.00248	-0.251	0.554	0.446	FA5	4.25	4.22	-0.0266	-0.626
CI	-0.0112	0.0812	-0.0327	0.138	-0.943	0.00354	-0.15	0.939	0.0609	FA5	4.25	4.44	0.186	4.39
TR	-0.0398	-0.096	-0.0515	0.725	-0.111	0.0172	-0.632	0.952	0.0484	FA4	4.25	4.04	-0.208	-4.88
WUE	-0.144	-0.0531	-0.0553	0.908	-0.0535	-0.0366	0.0519	0.857	0.143	FA4	4.25	4.63	0.374	8.8
LS	-0.0121	0.0822	-0.0346	0.138	-0.943	0.00444	-0.151	0.939	0.0606	FA5	4.25	4.06	-0.187	-4.4
Eigenvalues	3.02	2.55	1.93	1.68	1.58	1.11	1.09							
Variance (%)	18.9	15.9	12	10.5	9.89	6.93	6.79							
Cum. variance (%)	18.9	34.8	46.9	57.4	67.3	74.2	81							

Joint family														
H2	-0.833	-0.0517	-0.0895	-0.0846	0.0744	0.167	-0.00977	0.745	0.255	FA1	4.45	4.77	0.313	7.04
HS	-0.246	-0.0181	-0.0345	-0.057	-0.0283	0.667	-0.0845	0.519	0.481	FA6	3.91	4.02	0.102	2.61
D2	-0.95	-0.0229	-0.0409	0.00768	0.0282	0.0124	0.00977	0.905	0.0947	FA1	3.86	4.32	0.458	11.9
DS	-0.721	0.0617	0.0321	0.0652	-0.024	0.311	0.0081	0.627	0.373	FA1	4	4.28	0.283	7.08
LA	0.152	-0.0318	-0.0882	0.0459	-0.336	-0.667	-0.1	0.601	0.399	FA6	4.51	4.46	-0.052	-1.15
FW	-0.0581	-0.0373	-0.957	0.0438	0.157	-0.138	0.0304	0.967	0.0326	FA3	4.51	4.59	0.078	1.73
TW	0.0401	0.0222	0.976	-0.0511	0.00402	-0.117	-0.0209	0.972	0.0283	FA3	4.51	4.54	0.0284	0.63
LDW	-0.016	0.00518	0.114	-0.0548	-0.907	0.0947	0.0754	0.853	0.147	FA5	4.32	4.65	0.334	7.72
LMA	0.0913	-0.0159	0.0424	-0.011	-0.915	-0.36	-0.0108	0.977	0.0229	FA5	4.51	4.8	0.289	6.42
RWC	-0.0588	0.0202	-0.0133	0.0481	0.00554	0.866	-0.00814	0.756	0.244	FA6	4.32	4.35	0.0327	0.758
PN	0.00151	0.0542	-0.0505	0.0767	-0.0392	-0.0205	0.981	0.976	0.0242	FA7	4.51	4.58	0.0687	1.52
GA	0.0244	0.57	0.143	0.254	-0.0471	0.0346	-0.156	0.438	0.562	FA2	4.51	4.47	-0.0415	-0.921
CI	0.00238	-0.957	0.0331	0.13	-0.029	0.00118	-0.113	0.947	0.0533	FA2	4.51	4.65	0.137	3.03
TR	0.0135	-0.0558	-0.0183	0.726	0.0643	-0.0116	-0.649	0.957	0.0433	FA4	4.51	4.34	-0.168	-3.72
WUE	0.00766	-0.0318	-0.0944	0.965	0.0371	-0.0339	0.146	0.966	0.0343	FA4	4.51	4.83	0.318	7.05
LS	0.00196	-0.957	0.0326	0.13	-0.0282	-0.00011	-0.113	0.947	0.0527	FA2	4.32	4.19	-0.136	-3.15
Eigenvalues	3.01	2.37	2.04	1.83	1.58	1.19	1.13							
Variance (%)	18.8	14.8	12.7	11.4	9.91	7.45	7.04							
Cum. variance (%)	18.8	33.7	46.4	57.8	67.7	75.2	82.2							

*H2 *Plant height, *HS* Net increase in plant height, *D2 *Diameter,*DS* Net increase in base diameter, *LA* Leaf area, *FW* Leaf fresh Weight, *TW* Turgid weight, *LDW* Leaf dry weight, *LMA* Leaf Mass per Area, *RWC* Relative water content, *PN* Net photosynthesis rate, *GA* Stomatal conductance, *CI* Intercellular *CO*_*2*_ dioxide concentration, *TR* Transpiration, *WUE* Water use efficiency, *LS* Stomatal limitation

**Fig. 3:**
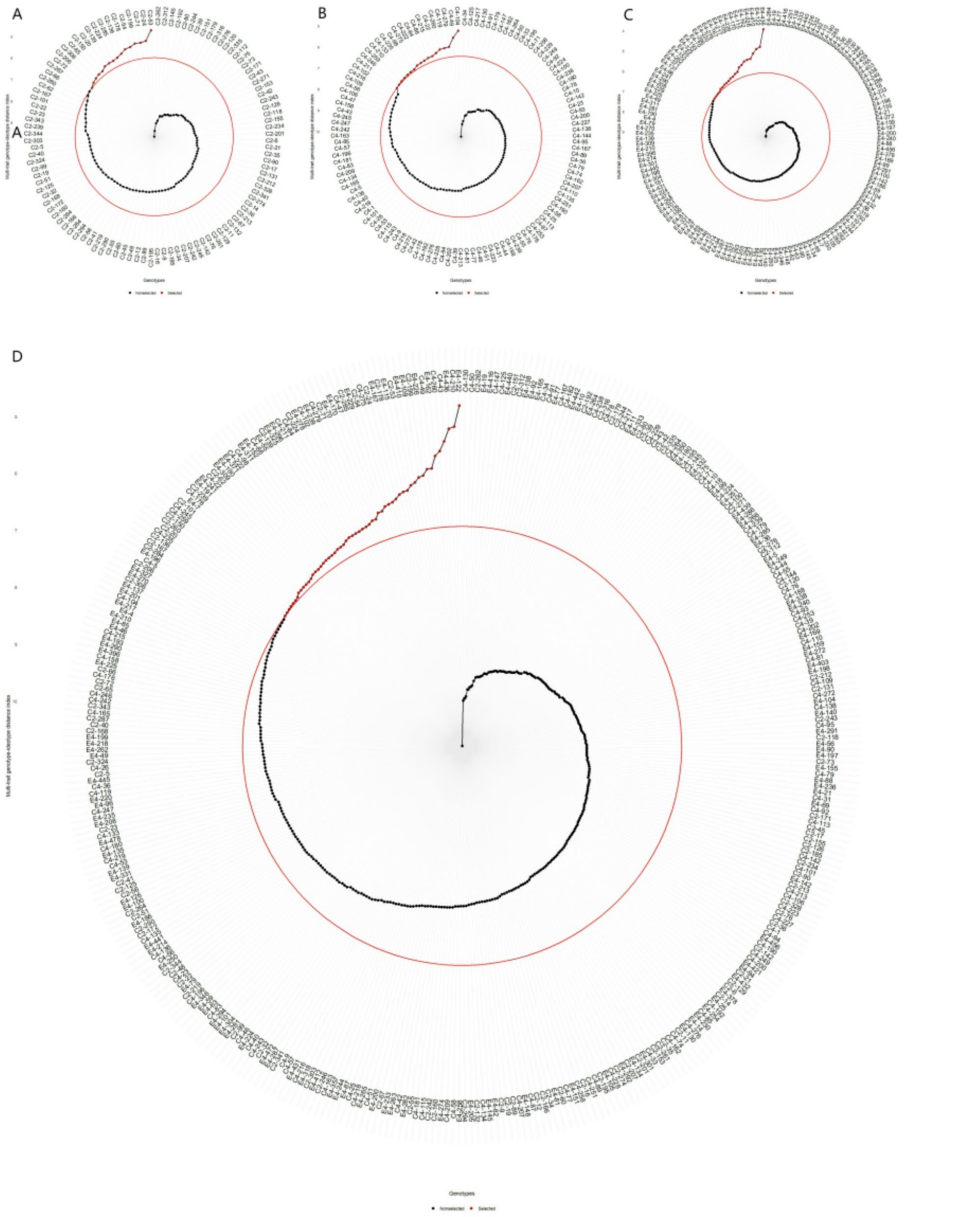
Genotype ranking and selection based on the MGIDI_BLUP for (**A**) family C2, ‘1-XY’×’N139’, (**B**) family C4, ‘1-XY’×’N188’, (**C**) family E4, ‘ZL-3’×’N188’, and (**D**) the joint family. Selected genotypes are highlighted in red, with a red circle indicating the selection threshold

Using a 20% selection intensity, the MGIDI_BLUP index identified 16, 18, 22, and 56 top genotypes from the ‘1‑XY’×’N139’, ‘1‑XY’×’N188’, ‘ZL‑3’×’N188’, and joint families, respectively (Fig. 3A-D). The genetic gains (SG%) based on this index showed a balanced and positive improvement across multiple trait categories (Table 5). The highest gains were consistently observed for growth traits, with D2 achieving up to 11.9% and H2 up to 7.04% in the joint family. Notably, unlike the SHI, the MGIDI_BLUP also facilitated positive gains for other traits, such as DS (up to 12.1%).

**Fig. 4:**
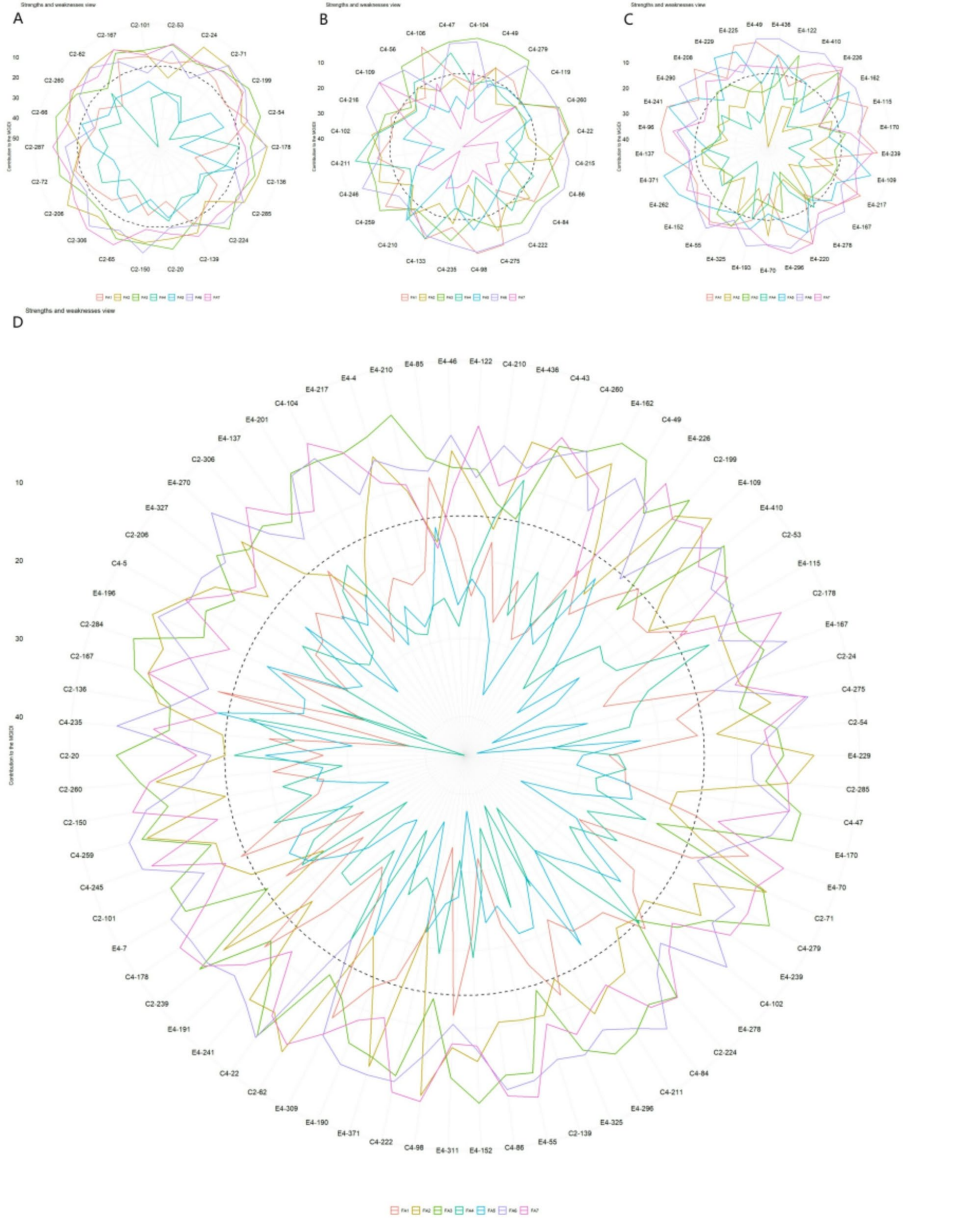
Strengths and weaknesses of the selected genotypes based on factor contributions to the MGIDI_BLUP for (**A**) family C2, ‘1-XY’×’N139’, (**B**) family C4, ‘1-XY’×’N188’, (**C**) family E4, ‘ZL-3’×’N188’, and (**D**) the joint family. For each selected genotype, the contribution of each factor is ranked visually from the most contributing (closest to the plot center) to the least contributing (farthest from the center). A smaller radial distance for a factor indicates that the traits within it are more similar to the ideotype

Radar plot analysis (Fig. 4A-D) provided a visual profile of the strengths and weaknesses of the selected genotypes relative to the ideotype for each factor. Although individual genotypes exhibited unique profiles, such as excelling in specific factors related to growth, leaf hydration, or photosynthesis, a consistent pattern emerged across families. Selected genotypes generally showed balanced performance closer to the ideotype across most factors, with no single factor being a predominant weakness for all, demonstrating the method’s efficacy in identifying genotypes with comprehensive trait improvement.

### Selection of hybrid progeny genotypes using MGIDI_LWindex

The MGIDI_LWindex, calculated from drought tolerance coefficients, also yielded a stable seven-factor solution across all families, explaining 74.4–77.7% of the total variance (Table 6). This analysis provided a distinct but complementary perspective on trait integration under drought stress compared to the indices based on genotypic values.

**Table 6:** EFA overlay retained factor eigenvalues, variances, loadings, and MGIDI_LWindex selection gains

‘1-XY’×’N139’														
**Traits**	**FA1**	**FA2**	**FA3**	**FA4**	**FA5**	**FA6**	**FA7**	**Communality**	**Uniquenesses**	**Factor**	**Xo**	**Xs**	**SD**	**SD%**
H2	-0.0024	0.0144	-0.779	-0.0176	-0.0504	0.118	-0.453	0.828	0.172	FA3	0.756	0.839	0.0829	11
HS	0.128	0.0819	-0.0935	0.0425	-0.0393	-0.00282	-0.897	0.839	0.161	FA7	0.436	0.499	0.0627	14.4
D2	-0.145	0.047	-0.877	-0.0967	0.0279	-0.111	0.119	0.829	0.171	FA3	0.87	0.964	0.0938	10.8
DS	-0.397	0.0836	-0.104	0.0857	0.0022	-0.436	0.0924	0.381	0.619	FA6	2.72	3.81	1.09	40.2
LA	0.103	0.26	-0.272	-0.0473	0.612	-0.0881	0.322	0.641	0.359	FA5	0.83	0.659	-0.171	-20.6
FW	0.952	0.0331	0.037	0.0437	-0.207	0.0638	-0.0605	0.961	0.0394	FA1	0.989	1.04	0.0492	4.98
TW	-0.956	0.0224	-0.078	-0.0541	0.088	-0.0501	0.0464	0.935	0.0646	FA1	1.15	1.19	0.0413	3.59
LDW	-0.318	-0.202	0.199	-0.00934	0.738	-0.139	-0.133	0.763	0.237	FA5	1.49	1.91	0.422	28.3
LMA	-0.173	-0.037	-0.00987	-0.0854	0.929	-0.107	0.0148	0.914	0.0863	FA5	2.21	3.36	1.16	52.3
RWC	0.0578	-0.454	0.0739	0.0775	0.254	0.117	0.0471	0.302	0.698	FA2	0.796	0.799	0.00301	0.377
PN	-0.0324	-0.884	-0.00782	0.125	-0.0597	-0.283	0.0653	0.887	0.113	FA2	0.824	0.879	0.0548	6.64
GA	0.0682	0.0522	-0.0198	0.52	-0.108	0.638	0.199	0.737	0.263	FA6	0.752	0.823	0.0715	9.51
CI	0.0215	0.14	-0.0811	-0.89	-0.00632	-0.0544	0.167	0.85	0.15	FA4	0.792	0.791	-0.000735	-0.0928
TR	0.01	0.837	-0.0083	-0.00738	0.113	-0.408	0.0404	0.883	0.117	FA2	0.779	0.667	-0.112	-14.4
WUE	-0.0355	0.101	0.0127	-0.000557	0.164	-0.891	0.057	0.836	0.164	FA6	1.36	1.92	0.557	40.9
LS	-0.0595	0.0642	-0.0394	-0.909	0.0744	0.0174	-0.0656	0.846	0.154	FA4	1.86	1.79	-0.0704	-3.78
Eigenvalues	3.24	2.28	1.67	1.56	1.35	1.29	1.05							
Variance (%)	20.3	14.2	10.4	9.75	8.41	8.07	6.55							
Cum. variance (%)	20.3	34.5	44.9	54.7	63.1	71.1	77.7							

‘1-XY’×’N188’														
H2	-0.221	-0.109	-0.0247	-0.816	0.124	-0.00324	0.0104	0.742	0.258	FA4	0.769	0.892	0.122	15.9
HS	-0.00984	0.201	-0.0528	-0.0778	0.473	0.00423	-0.116	0.287	0.713	FA5	0.47	0.477	0.00767	1.63
D2	-0.00447	0.0175	-0.0463	-0.907	-0.0901	-0.0442	-0.0826	0.841	0.159	FA4	0.904	1.03	0.123	13.6
DS	0.204	0.167	-0.307	-0.163	-0.556	-0.161	-0.0103	0.525	0.475	FA5	3.88	2.82	-1.07	-27.5
LA	0.0608	-0.114	-0.0901	0.00331	0.0843	-0.0946	0.823	0.719	0.281	FA7	0.555	0.538	-0.0161	-2.9
FW	-0.222	-0.00741	-0.0163	-0.0307	0.0134	-0.963	0.0742	0.983	0.0169	FA6	1.04	0.993	-0.0469	-4.51
TW	0.102	-0.00588	-0.000809	0.0177	-0.0306	0.984	-0.00653	0.98	0.0197	FA6	1.19	1.11	-0.0855	-7.18
LDW	0.902	-0.0102	0.132	0.129	-0.051	0.199	-0.175	0.92	0.0796	FA1	1.47	2.06	0.587	40
LMA	0.925	-0.0644	0.0997	0.108	0.0116	0.149	0.179	0.936	0.0642	FA1	2.96	4.31	1.35	45.8
RWC	0.077	-0.159	-0.0978	-0.156	0.353	-0.0202	-0.595	0.544	0.456	FA7	0.826	0.833	0.00781	0.946
PN	0.112	0.0517	0.911	0.00265	0.106	-0.0292	-0.00662	0.857	0.143	FA3	0.815	0.769	-0.0461	-5.65
GA	0.0148	0.492	0.0488	0.244	-0.253	-0.0548	-0.391	0.524	0.476	FA2	0.76	0.797	0.0371	4.89
CI	-0.0393	-0.891	0.00805	-0.00381	-0.0994	-0.0393	0.11	0.818	0.182	FA2	0.786	0.789	0.00264	0.336
TR	-0.131	-0.00301	-0.876	-0.0765	0.352	-0.0399	0.039	0.918	0.0821	FA3	0.777	0.677	-0.101	-13
WUE	0.0696	0.0135	-0.196	-0.00768	0.769	-0.139	0.0825	0.661	0.339	FA5	1.31	1.46	0.149	11.4
LS	0.109	-0.85	-0.0337	-0.0182	-0.0879	0.0195	-0.154	0.768	0.232	FA2	1.92	1.75	-0.172	-8.97
Eigenvalues	2.9	2.03	1.81	1.56	1.35	1.22	1.15							
Variance (%)	18.1	12.7	11.3	9.77	8.42	7.59	7.19							
Cum. variance (%)	18.1	30.8	42.2	51.9	60.4	68	75.1							

‘ZL-3’×’N188’														
H2	-0.155	0.0421	-0.0902	0.0205	-0.866	0.0147	-0.0835	0.791	0.209	FA5	0.834	1.12	0.283	34
HS	-0.131	0.0112	-0.296	0.0199	-0.589	-0.139	-0.0825	0.478	0.522	FA5	3.6	9.2	5.59	155
D2	0.0738	0.00787	0.211	0.021	-0.736	0.146	0.0173	0.613	0.387	FA5	0.906	1.05	0.148	16.4
DS	0.632	0.167	-0.145	-0.0934	-0.0303	-0.061	-0.305	0.554	0.446	FA1	3.06	4.08	1.02	33.2
LA	-0.204	0.00152	-0.0507	0.14	0.0104	-0.00675	-0.775	0.664	0.336	FA7	0.633	0.551	-0.0812	-12.8
FW	0.121	-0.978	-0.0492	0.0549	0.0322	-0.00826	-0.0328	0.98	0.0203	FA2	1.08	1.13	0.0452	4.18
TW	-0.0351	0.983	0.0485	-0.0518	-0.0239	0.000261	0.0773	0.979	0.021	FA2	1.23	1.26	0.0328	2.66
LDW	-0.851	0.218	-0.0814	-0.0229	-0.134	-0.0352	-0.0771	0.804	0.196	FA1	1.55	1.77	0.221	14.3
LMA	-0.834	0.139	-0.101	0.00567	-0.132	-0.0204	-0.412	0.914	0.0865	FA1	2.74	3.6	0.857	31.3
RWC	0.0804	-0.101	0.0836	-0.0502	-0.0985	0.0435	-0.627	0.431	0.569	FA7	0.828	0.843	0.015	1.81
PN	-0.0148	0.0987	0.928	0.0189	0.0508	0.134	-0.0307	0.893	0.107	FA3	0.817	0.779	-0.0374	-4.57
GA	-0.187	-0.025	0.00575	0.538	0.0899	-0.484	0.149	0.589	0.411	FA4	0.754	0.815	0.0615	8.17
CI	-0.00264	0.0798	0.0233	-0.922	0.0377	-0.0194	0.0501	0.862	0.138	FA4	0.788	0.799	0.0113	1.44
TR	-0.045	0.00494	-0.82	0.0329	-0.0353	0.492	0.0261	0.92	0.0801	FA3	0.756	0.712	-0.0438	-5.8
WUE	-0.0515	-0.00207	-0.0514	0.0736	-0.00669	0.921	-0.000163	0.859	0.141	FA6	1.34	1.43	0.0927	6.92
LS	-0.0126	0.0154	-0.0164	-0.878	0.0551	-0.0848	0.0728	0.788	0.212	FA4	1.92	1.85	-0.0689	-3.59
Eigenvalues	2.62	2.25	1.94	1.52	1.45	1.25	1.1							
Variance (%)	16.4	14	12.1	9.52	9.07	7.83	6.85							
Cum. variance (%)	16.4	30.4	42.5	52	61.1	68.9	75.7							

Joint family														
H2	-0.000408	-0.0441	-0.0207	0.893	-0.0161	-0.0159	0.00157	0.801	0.199	FA4	0.791	1.03	0.242	30.7
HS	-0.0177	-0.173	0.0528	0.592	-0.249	0.0946	-0.046	0.457	0.543	FA4	1.68	5.2	3.52	209
D2	0.0956	0.0954	-0.0374	0.687	0.213	-0.131	0.243	0.613	0.387	FA4	0.895	1.06	0.164	18.3
DS	0.232	-0.0988	-0.0219	-0.115	0.352	-0.0155	0.471	0.423	0.577	FA7	3.22	2.6	-0.628	-19.5
LA	-0.0404	-0.13	-0.0262	0.00286	-0.221	-0.104	0.742	0.629	0.371	FA7	0.665	0.587	-0.0776	-11.7
FW	-0.964	-0.0358	0.0276	-0.034	0.201	0.0104	-0.0363	0.974	0.026	FA1	1.04	1.07	0.0259	2.49
TW	0.976	0.0188	-0.0407	0.0334	-0.0966	-0.0121	-0.0139	0.965	0.0352	FA1	1.2	1.2	0.00778	0.651
LDW	0.231	0.0588	-0.000724	0.00253	-0.862	-0.016	-0.00561	0.8	0.2	FA5	1.51	1.86	0.356	23.6
LMA	0.128	-0.00609	-0.0319	0.0309	-0.886	-0.0403	0.316	0.905	0.0949	FA5	2.66	3.7	1.04	39.3
RWC	-0.0348	0.117	0.0495	0.173	-0.0917	0.067	0.593	0.412	0.588	FA7	0.818	0.832	0.0133	1.63
PN	0.0454	0.934	0.0713	-0.0636	-0.044	-0.173	-0.0274	0.917	0.0832	FA2	0.818	0.842	0.0234	2.86
GA	-0.0398	-0.0446	0.497	-0.0523	-0.053	0.567	-0.123	0.593	0.407	FA6	0.755	0.791	0.0361	4.78
CI	0.0159	-0.0288	-0.916	-0.00964	0.00921	-0.0346	-0.0295	0.842	0.158	FA3	0.789	0.79	0.00102	0.129
TR	-0.00318	-0.851	0.0302	0.0727	0.0278	-0.438	0.0219	0.924	0.0763	FA2	0.77	0.727	-0.0427	-5.55
WUE	0.000357	-0.0895	0.0816	-0.00469	-0.0686	-0.921	-0.0322	0.868	0.132	FA6	1.34	1.45	0.11	8.2
LS	0.0361	-0.0293	-0.884	-0.00556	-0.0422	0.0448	-0.024	0.788	0.212	FA3	1.9	1.86	-0.0416	-2.19
Eigenvalues	2.59	2.09	1.87	1.53	1.46	1.26	1.12							
Variance (%)	16.2	13.1	11.7	9.55	9.11	7.88	6.99							
Cum. variance (%)	16.2	29.3	40.9	50.5	59.6	67.5	74.4							

*H2 *Plant height, *HS* Net increase in plant height, *D2 *Diameter, *DS* Net increase in base diameter, *LA* Leaf area, *FW* Leaf fresh Weight, *TW* Turgid weight, *LDW* Leaf dry weight, *LMA* Leaf Mass per Area, *RWC* Relative water content, *PN* Net photosynthesis rate, *GA* Stomatal conductance, *CI* Intercellular *CO*_*2*_ dioxide concentration, *TR* Transpiration, *WUE* Water use efficiency, *LS* Stomatal limitation

**Fig. 5:**
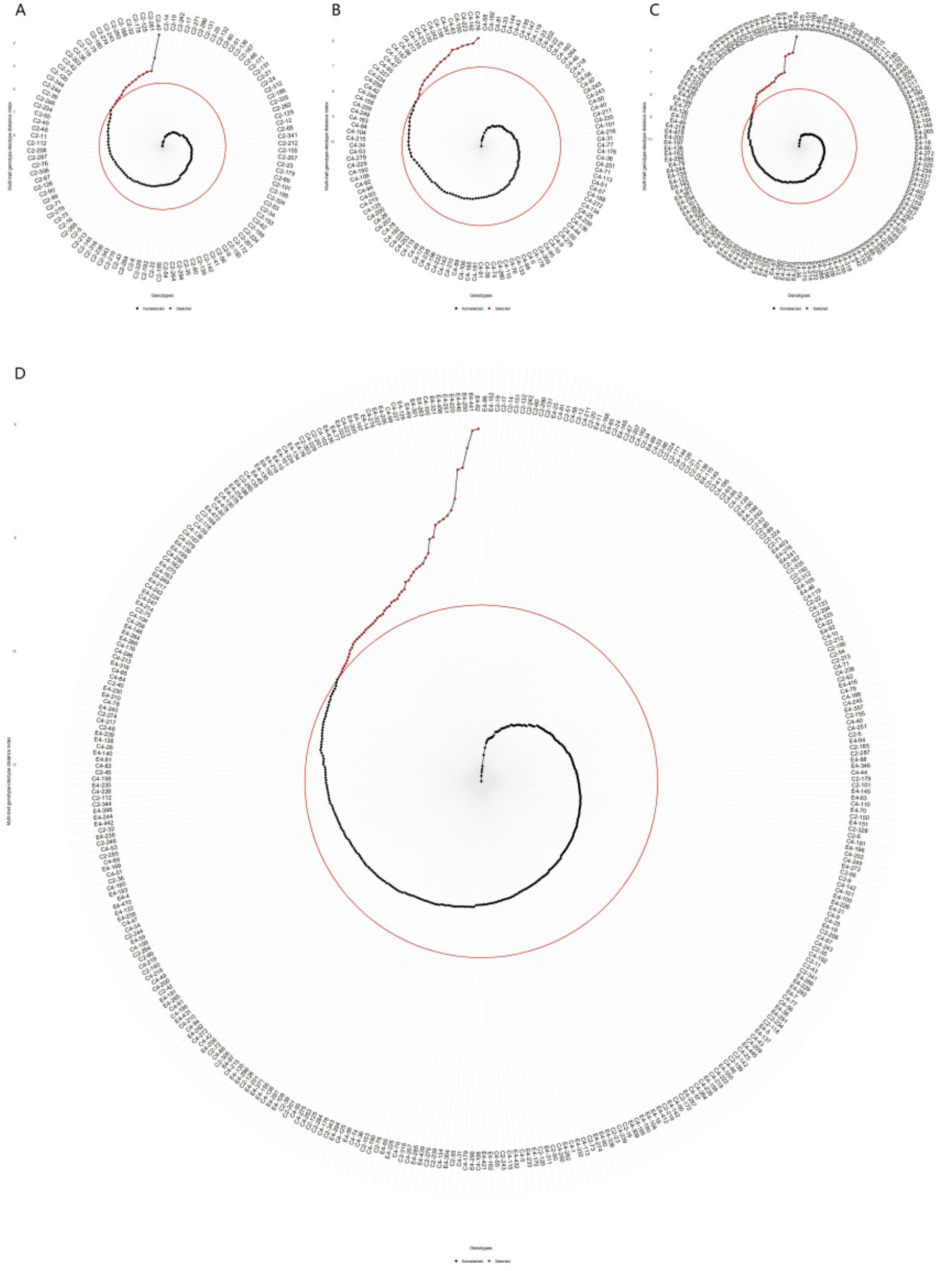
Genotype ranking and selection based on the MGIDI_LWindex for (**A**) family C2, ‘1-XY’×’N139’, (**B**) family C4, ‘1-XY’×’N188’, (**C**) family E4, ‘ZL-3’×’N188’, and (**D**) the joint family. Selected genotypes are highlighted in red, with a red circle indicating the selection threshold

Applying a 20% selection intensity based on the MGIDI_LWindex identified 16, 18, 22, and 56 superior genotypes from the ‘1‑XY’×’N139’, ‘1‑XY’×’N188’, ‘ZL‑3’×’N188’, and joint families, respectively (Fig. 5A-D). Representative selections included C2‑40, C4‑276, E4‑250, and E4‑82. The MGIDI_LWindex prioritized genotypes with high drought-adaptive capacity, resulting in exceptionally high genetic gains for key growth maintenance traits under stress (Table 6). Notably, HS showed a dramatic gain of 209% in the joint family selection, while H2 and D2 also achieved substantial improvements (up to 30.7% and 18.3%, respectively). This pattern underscores the index’s effectiveness in screening for genotypes that not only survive but also maintain growth under drought conditions.

**Fig. 6:**
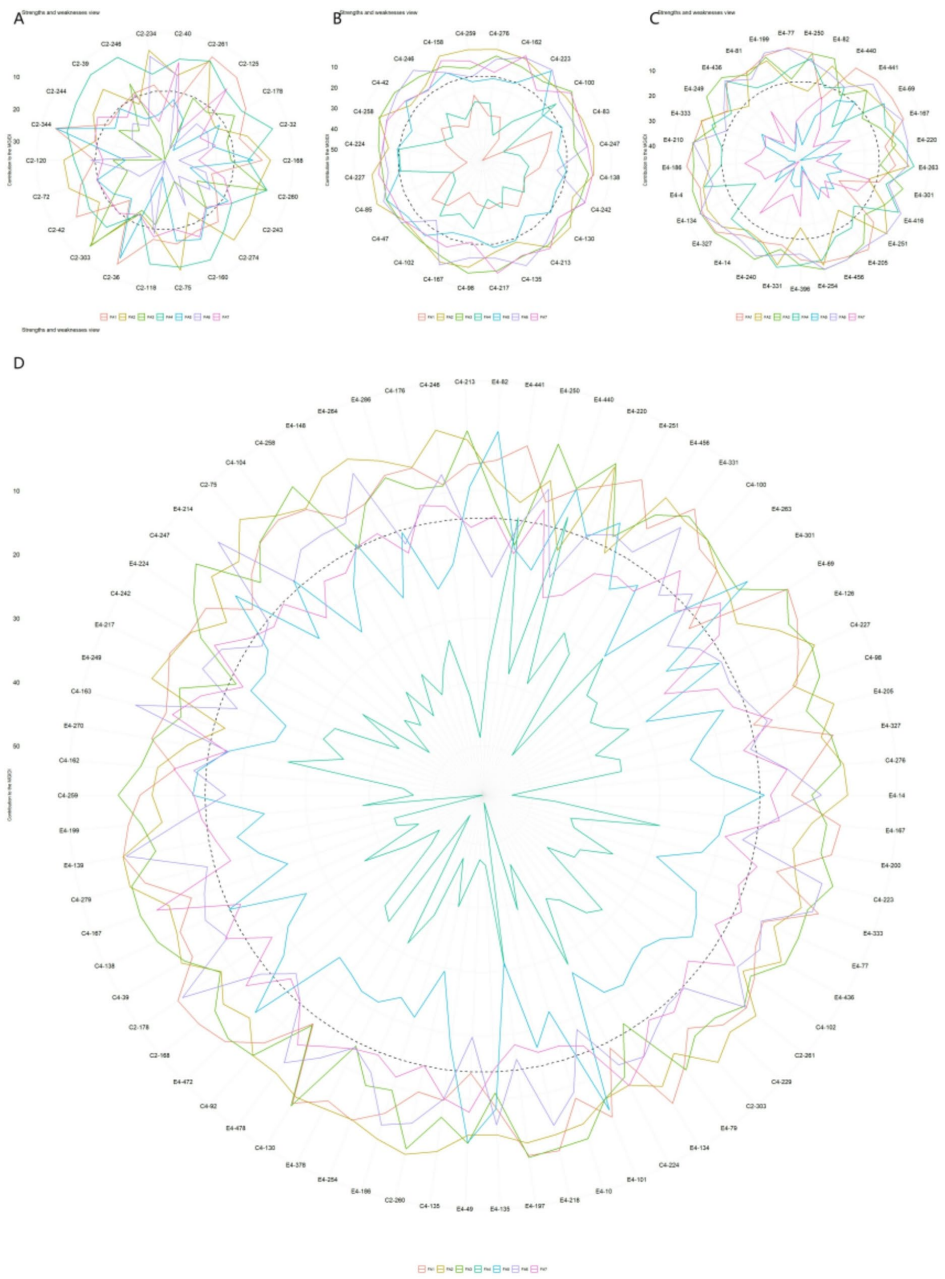
Strengths and weaknesses of the selected genotypes based on factor contributions to the MGIDI_LWindex for (**A**) family C2, ‘1-XY’×’N139’, (**B**) family C4, ‘1-XY’×’N188’, (**C**) family E4, ‘ZL-3’×’N188’, and (**D**) the joint family. For each selected genotype, the contribution of each factor is ranked visually from the most contributing (closest to the plot center) to the least contributing (farthest from the center). A smaller radial distance for a factor indicates that the traits within it are more similar to the ideotype

Analysis of genotype profiles via radar plots (Fig. 6A-D) further elucidated the drought-adaptive strategies of the selected genotypes. The performance patterns were family-specific, but overall, selected genotypes showed a tendency to maintain strengths in factors related to leaf water status (e.g., FW, TW, RWC) and photosynthetic stability under stress, aligning with the index’s selection criterion.

### Correlation analysis and Venn diagram results

**Fig. 7:**
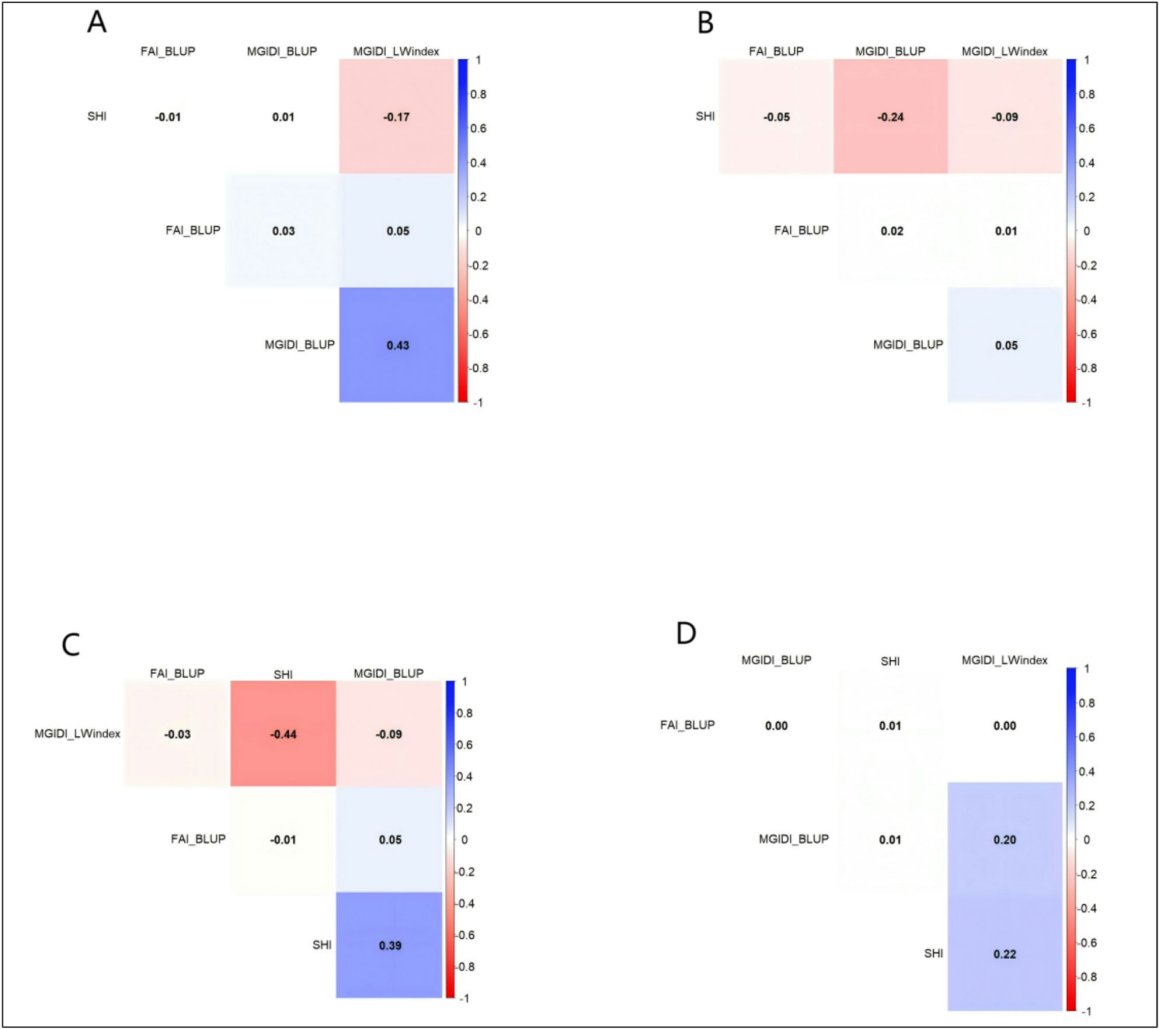
Heatmaps of Spearman’s rank correlation coefficients among selection indices for (**A**) family C2, ‘1-XY’×’N139’, (**B**) family C4, ‘1-XY’×’N188’, (**C**) family E4, ‘ZL-3’×’N188’, and (**D**) the joint family. Color gradients indicate the strength and direction of the associations

To assess the consistency among selection methods, we analyzed the Spearman’s rank correlations between the four indices (SHI, FAI-BLUP, MGIDI_BLUP, MGIDI_LWindex). Overall, correlations were weak across all families (Fig. 7A-D). Most correlation coefficients had an absolute value below 0.2, indicating that each index provides largely independent ranking information. The strongest associations were moderate and family-specific, such as a positive correlation between MGIDI_BLUP and MGIDI_LWindex in the ‘1‑XY’×’N139’ family (r = 0.43) and a negative correlation between SHI and MGIDI_LWindex in the ‘ZL‑3’×’N188’ family (*r* = − 0.44).

**Fig. 8:**
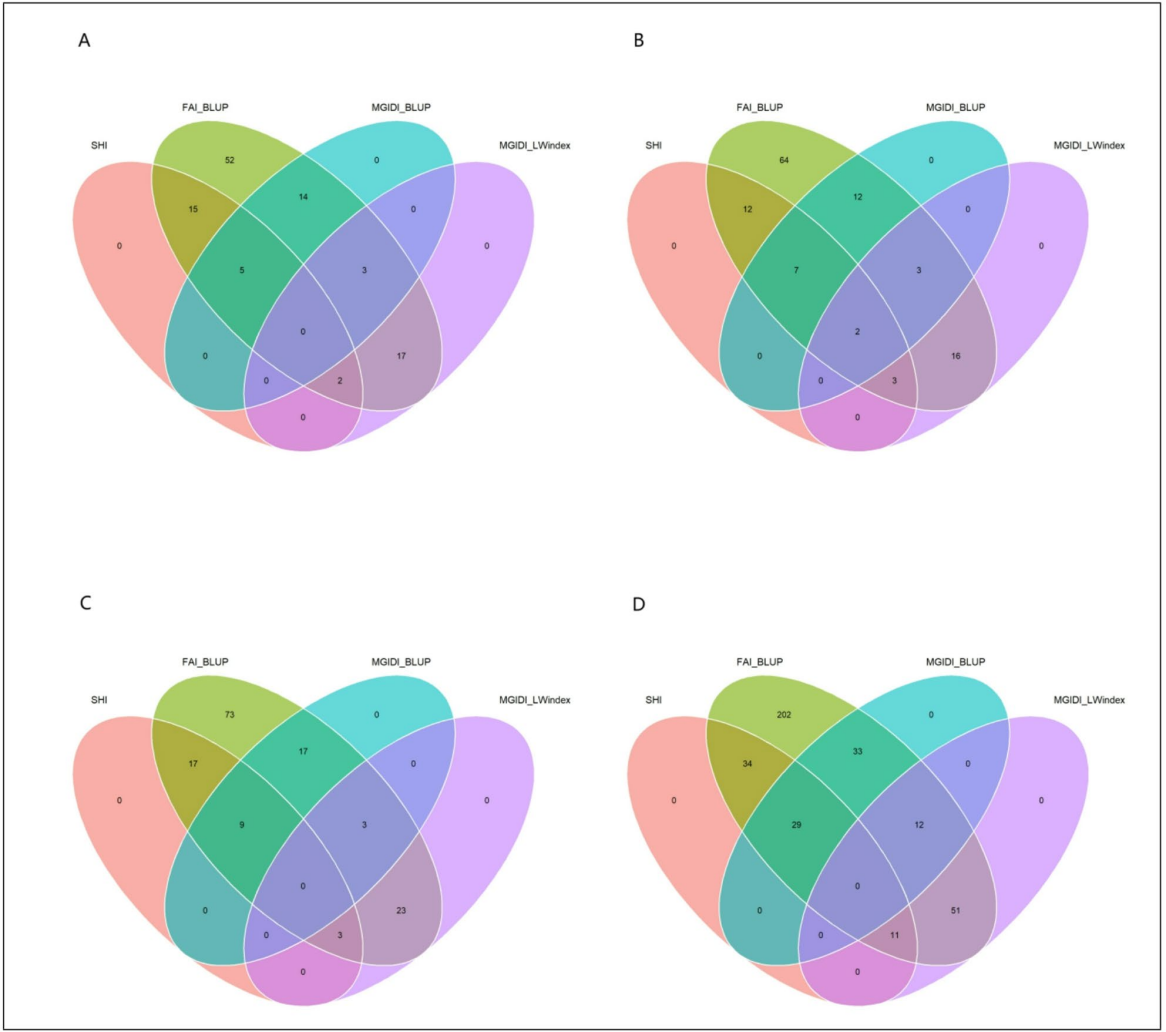
Venn diagrams showing the overlap of clones selected by multiple indices (SHI, FAI_BLUP, MGIDI_BLUP, MGIDI_LWindex) for (**A**) family C2, ‘1-XY’×’N139’, (**B**) family C4, ‘1-XY’×’N188’, (**C**) family E4, ‘ZL-3’×’N188’, and (**D**) the joint family. See Supplementary Table S1 for corresponding genotype lists

Venn diagram analysis (Fig. 8A-D) identified genotypes that were consistently selected across multiple indices. The number within each segment of the diagrams indicates the count of genotypes in that category (the complete list of genotypes selected by two or more indices is provided in Supplementary Table S1). While the specific genotypes varied by family, several demonstrated robust performance. For instance, in the joint family, E4‑70 and E4‑309 (overlap of SHI, FAI-BLUP, and MGIDI_BLUP) were highly ranked by three indices. Notably, in the ‘1‑XY’×’N188’ family, genotypes C4‑246 and C4‑102 (overlap of all four indices) were selected by every evaluation criterion. These results confirm that different indices can select distinct sets of genotypes, while also highlighting a core group of superior genotypes with consistent multi-trait merit.

### Weighted Rank Aggregation (WRA) based on Rranker Accuracies (RA)

To reconcile the divergent rankings from the four selection indices, we applied Weighted Rank Aggregation (WRA) to derive a consensus ranking. The reliability-based weights assigned to each index differed markedly among families, reflecting their distinct genetic backgrounds. In the ‘1‑XY’×’N139’ family, FAI-BLUP received the highest weight (0.555), followed by SHI (0.263), MGIDI_BLUP (0.106), and MGIDI_LWindex (0.077). For the ‘1‑XY’×’N188’ family, the weights were 0.499 for FAI-BLUP, 0.267 for MGIDI_LWindex, 0.129 for MGIDI_BLUP, and 0.105 for SHI. In the ‘ZL‑3’×’N188’ family, FAI-BLUP was assigned the greatest weight (0.695), with lower weights for MGIDI_LWindex (0.118), SHI (0.112), and MGIDI_BLUP (0.074). Conversely, in the joint family analysis, SHI carried the predominant weight (0.899), while the weights for FAI-BLUP, MGIDI_BLUP, and MGIDI_LWindex were minimal (0.042, 0.037, and 0.021, respectively).

**Fig. 9:**
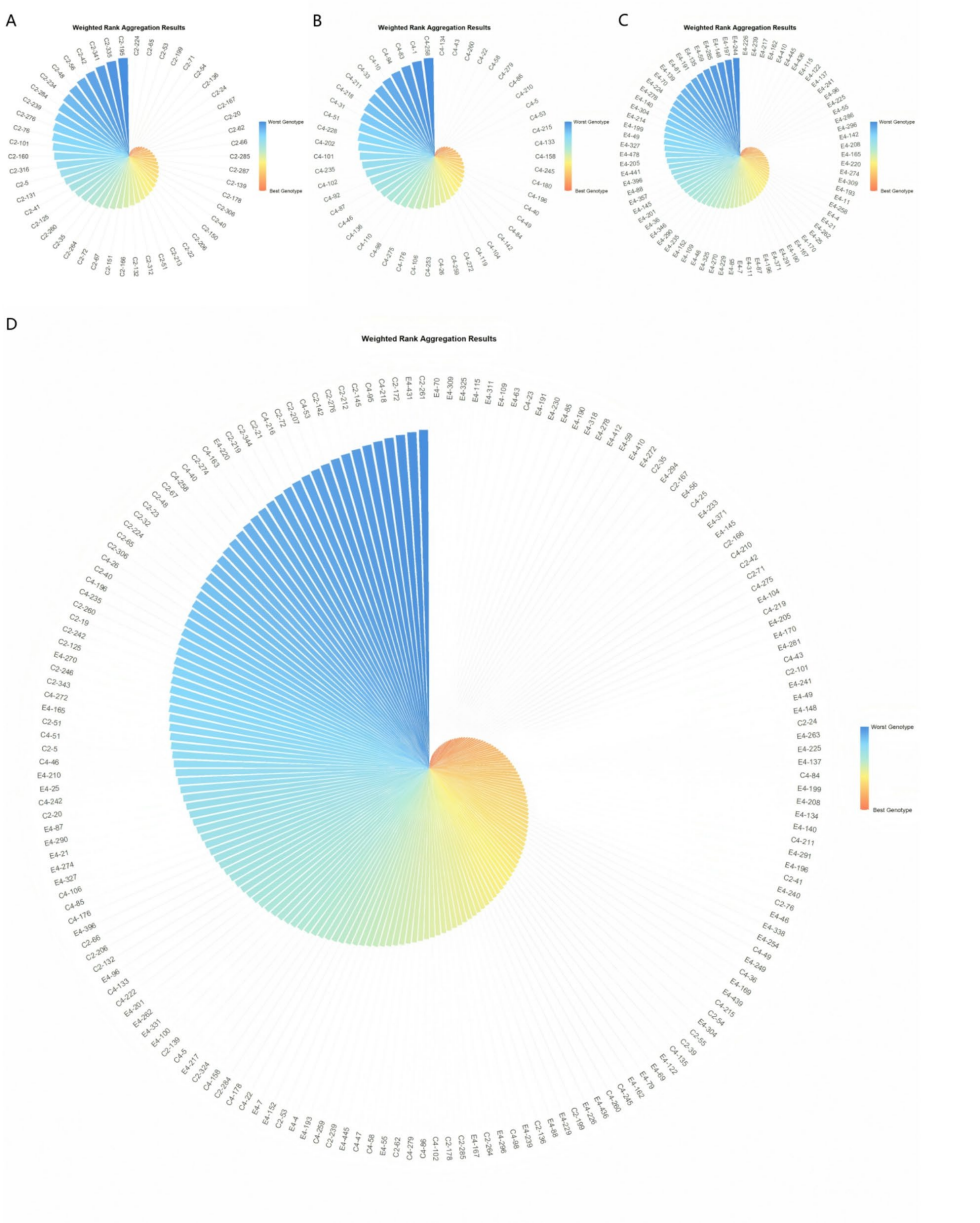
Weighted rank aggregation (WRA) plots for the top-ranked genotypes based on SHI, FAI_BLUP, MGIDI_BLUP, and MGIDI_LWindex for (**A**) family C2, ‘1-XY’×’N139’, (**B**) family C4, ‘1-XY’×’N188’, (**C**) family E4, ‘ZL-3’×’N188’, and (**D**) the joint family

The resulting WRA rankings provided an integrated assessment for each family (Fig. 9A-D). Lowest weight scores are considered to correspond to the best genotypes compared to the highest weight scores. The top-ranked genotypes from this consensus analysis were: C2‑224, C2‑65, and C2‑53 from ‘1‑XY’×’N139’; C4‑134, C4‑43, and C4‑260 from ‘1‑XY’×’N188’; E4‑226, E4‑239, and E4‑217 from ‘ZL‑3’×’N188’; and E4‑70, E4‑309, and E4‑325 from the joint family (see Supplementary Table S2 for complete rankings and scores).

These WRA-selected genotypes represent robust candidates that synthesize the selection signals from all four methods. Notably, several genotypes identified here, such as E4‑70 and C4‑246, also appeared among the top performers in the multi-index Venn diagram analysis, reinforcing their breeding value.

## Discussion

This study demonstrates that integrating multivariate selection indices via Weighted Rank Aggregation (WRA) provides a robust strategy for drought-tolerant poplar breeding. The significant genetic and genotype-by-environment (G×E) variance observed for most traits (Table 1) confirms a substantial genetic basis for selection, consistent with previous findings in poplar [57, 58]. However, effectively harnessing this diversity requires methods that can address trait interdependence and environmental variability—a core challenge in modern breeding [59].

The differential performance of the indices reflects their distinct methodological foundations and inherent biological trade-offs. The Smith-Hazel index (SHI), a classical tool for weighted selection [47, 48], delivered strong directional gains for target growth traits (H2, D2), confirming its efficacy for genetic improvement as seen in crops like tomato and rice [18, 60]. However, its sensitivity to multicollinearity within individual families, leading to paradoxical selections, underscores a key limitation: fixed economic weights struggle to capture dynamic physiological resource reallocation under drought, where investments in growth, leaf mass, and water conservation often compete [61].

Advanced indices like FAI-BLUP and MGIDI mitigate this by modeling the trait covariance structure. FAI-BLUP, which integrates factor analysis with BLUP to separate genetic from environmental effects [62], enabled more balanced selection across trait categories. Its ability to identify genotypes with coordinated leaf and growth improvements aligns with its successful application for balanced genetic gain in crops like barley and soybean [63–65], and mirrors the utility of multivariate models in selecting cacao genotypes stable across irrigation regimes [66]. The MGIDI index, by circumventing subjective weight assignment through factor analysis [51], offers a distinct advantage for holistic selection. Our results affirm its effectiveness for balanced trait improvement, as previously documented in upland cotton, sesame, and rice [67–69]. Notably, the MGIDI_LWindex variant, focused on drought-adaptive coefficients, excelled in selecting for growth maintenance under stress (e.g., exceptional HS gain), directly targeting a key component of drought resilience. This approach resonates with strategies in wheat breeding that employ stress tolerance indices to identify genotypes with superior yield maintenance under drought [14], and parallels the successful use of MGIDI for selecting climate-smart rice genotypes under water-saving cultivation [16].

The low correlations among indices are not a flaw but a validation of their complementary nature. Each index encapsulates a different ideal: SHI emphasizes weighted performance, FAI-BLUP emphasizes stability and balance, and MGIDI emphasizes proximity to an ideotype. This divergence confirms that a single statistical model cannot fully represent the multifaceted nature of drought tolerance [51, 70]. Consequently, reliance on any single index may overlook valuable genotypes, a principle highlighted in broader plant breeding frameworks that advocate for multi-trait, multi-method selection to improve reliability [27, 71, 72].

Therefore, the adoption of the Weighted Rank Aggregation (WRA) method in this study represents a novel and necessary synthesis for poplar breeding. By assigning data-driven weights to each index based on family-specific reliability, WRA moves beyond declaring a single “best” method. It provides a flexible, consensus-building tool that respects the context-dependent value of different selection models. This integrative approach, while established in other fields, has been rarely applied in forest tree breeding, particularly for drought tolerance selection. Our work demonstrates its utility as a robust decision-making framework that can synthesize complex, multi-dimensional data, a capability crucial for developing resilient cultivars in the face of climate change [16]. This aligns with advanced data synthesis strategies in plant breeding [73–75].

Notably, several genotypes were consistently selected across multiple indices and the final WRA ranking, including C2‑65, C2‑224, and C2‑20 from the ‘1‑XY’×’N139’ family; C4‑210 from ‘1‑XY’×’N188’; E4‑115, E4‑410, and E4‑436 from ‘ZL‑3’×’N188’; and E4‑70, E4‑309, E4‑325, E4‑311, E4‑109, and E4‑191 from the joint family. These genotypes exhibited optimal and balanced performance across growth, leaf, and photosynthetic physiological traits. This consistency not only highlights their breeding potential but also demonstrates the efficacy of the integrated multi-index and WRA framework for robust selection.

## Conclusion

This study establishes an effective, integrated selection strategy for identifying drought-tolerant *Populus simonii* × *P. nigra* progenies at the seedling stage. By synthesizing the complementary information from four multivariate selection indices (SHI, FAI-BLUP, MGIDI_BLUP, and MGIDI_LWindex) through Weighted Rank Aggregation (WRA), we identified a robust set of superior genotypes. Key selections include C2‑65, C2‑224, E4‑115, E4‑410, E4‑70, and E4‑309, which consistently demonstrated an optimal balance of drought-adaptive capacity, growth potential, and physiological efficiency across multiple evaluation criteria.

The primary methodological contribution of this work is the demonstrated application of the WRA framework to reconcile divergent selection outcomes in forest tree breeding. This approach moves beyond the debate over a single optimal index by providing a data-driven, consensus-building tool that is adaptable to specific genetic backgrounds and breeding objectives.

The selected genotypes offer immediate value as elite candidates for advanced testing and population development in drought-prone environments. For breeding programs, we recommend adopting a two-stage selection pipeline: (1) primary screening using a balanced index like MGIDI to identify broadly superior individuals, followed by (2) consensus refinement using WRA for final selection, especially when dealing with multiple families or complex trait trade-offs.

Future work should focus on validating the field performance and stability of these selections across diverse sites and longer growth cycles. While this study provides a robust phenotypic selection framework, the underlying genetic architecture and molecular mechanisms contributing to the observed drought tolerance in these elite progenies remain to be elucidated. Future investigations integrating genomics, transcriptomics, or other multi-omics approaches could uncover the key genes and pathways involved, potentially refining the WRA weighting algorithm with prior biological knowledge and enhancing the predictive power of selection models in perennial crops.

## Supplementary Information

Supplementary Material 1.

## Data Availability

All data generated or analyzed during this study is included in this article.
